# Tol-Pal System and Rgs Proteins Interact to Promote Unipolar Growth and Cell Division in Sinorhizobium meliloti

**DOI:** 10.1128/mBio.00306-20

**Published:** 2020-06-30

**Authors:** Elizaveta Krol, Hamish C. L. Yau, Marcus Lechner, Simon Schäper, Gert Bange, Waldemar Vollmer, Anke Becker

**Affiliations:** a Center for Synthetic Microbiology (SYNMIKRO), Philipps-Universität Marburg, Marburg, Germany; b Department of Biology, Philipps-Universität Marburg, Marburg, Germany; c Center for Bacterial Cell Biology, Biosciences Institute, Newcastle University, Newcastle upon Tyne, United Kingdom; d Faculty of Chemistry, Philipps-Universität Marburg, Marburg, Germany; John Innes Centre; University of California, Berkeley

**Keywords:** *Alphaproteobacteria*, *Rhizobiales*, cell division, cell growth, elongasome, peptidoglycan synthesis

## Abstract

Sinorhizobium meliloti is an alphaproteobacterium belonging to the *Rhizobiales*. Bacteria from this order elongate their cell wall at the new cell pole, generated by cell division. Screening for protein interaction partners of the previously characterized polar growth factors RgsP and RgsM, we identified the inner membrane components of the Tol-Pal system (TolQ and TolR) and novel Rgs (rhizobial growth and septation) proteins with unknown functions. TolQ, Pal, and all Rgs proteins, except for RgsE, were indispensable for S. meliloti cell growth. Six of the Rgs proteins, TolQ, and Pal localized to the growing cell pole in the cell elongation phase and to the septum in predivisional cells, and three Rgs proteins localized to the growing cell pole only. The putative FtsN-like protein RgsS contains a conserved SPOR domain and is indispensable at the early stages of cell division. The components of the Tol-Pal system were required at the late stages of cell division. RgsE, a homolog of the Agrobacterium tumefaciens growth pole ring protein GPR, has an important role in maintaining the normal growth rate and rod cell shape. RgsD is a periplasmic protein with the ability to bind peptidoglycan. Analysis of the phylogenetic distribution of the Rgs proteins showed that they are conserved in *Rhizobiales* and mostly absent from other alphaproteobacterial orders, suggesting a conserved role of these proteins in polar growth.

## Abstract (precis)

Bacterial cell proliferation involves cell growth and septum formation followed by cell division. For cell growth, bacteria have evolved different complex mechanisms. The most prevalent growth mode of rod-shaped bacteria is cell elongation by incorporating new peptidoglycans in a dispersed manner along the sidewall. A small share of rod-shaped bacteria, including the alphaproteobacterial *Rhizobiales*, grow unipolarly. Here, we identified and initially characterized a set of Rgs (rhizobial growth and septation) proteins, which are involved in cell division and unipolar growth of Sinorhizobium meliloti and highly conserved in *Rhizobiales*. Our data expand the knowledge of components of the polarly localized machinery driving cell wall growth and suggest a complex of Rgs proteins with components of the divisome, differing in composition between the polar cell elongation zone and the septum.

## INTRODUCTION

The life cycle of unicellular bacteria includes genome replication and approximate duplication of cell size followed by cell division. Increasing the cell volume relies on cell wall growth, which requires elongation of the peptidoglycan (PG) sacculus. Most of the rod-shaped bacteria elongate by incorporating new PGs in a dispersed manner along the sidewall, with the filaments of actin homolog MreB providing a scaffold for the elongasome PG biosynthesis machinery (1, 2). The cell division process relies on constriction of the Z-ring that consists of the tubulin homolog FtsZ, a core component of the dynamic divisome complex (3). The Escherichia coli divisome comprises more than twenty different proteins, including SPOR domain protein FtsN and the envelope-spanning Tol-Pal complex (4, 5). The latter consists of the inner membrane protein TolQ, the inner membrane-anchored periplasmic proteins TolR and TolA, the outer membrane-anchored protein Pal, and the Pal-associated periplasmic protein TolB (5, 6). Both elongasome and divisome include PG synthases and hydrolases, whose activities are tightly regulated in time and space to maintain cell shape and ensure cell wall integrity (6).

Whereas FtsZ-mediated cell division is conserved among most of the bacterial phyla (7), MreB-dependent cell elongation is less ubiquitous. In Gram-positive streptomycetes and corynebacteria in the phylum *Actinobacteria*, the polar protein DivIVA fulfills a function in scaffolding PG biosynthesis during cell elongation, which results in bipolar cell growth in rod-shaped species and apical growth at hyphal tips in filamentous species (8–10). The Gram-negative alphaproteobacterial order *Rhizobiales* includes species that lost MreB in the course of evolution (11). Unipolar cell elongation in rod-shaped *Rhizobiales*, characterized by insertion of new cell wall material at the new cell pole generated by cell division, was reported for several members of this order, such as the plant pathogen Agrobacterium tumefaciens, the plant symbiont Sinorhizobium meliloti, and the animal pathogen Brucella abortus (11–13). Despite ample evidence for polar cell growth in *Rhizobiales*, the scaffolding and regulatory factors governing this process remain largely unknown.

In A. tumefaciens, the conserved divisome proteins FtsZ, FtsA, and FtsW were suggested to regulate transition from polar growth to cell division (14, 15). Cells depleted for these proteins produce branches originating from the septal site (14). The exceptionally large growth pole ring protein GPR, which localizes to the growing cell pole, was shown to be required for normal polar growth and rod cell morphology in A. tumefaciens (16). Its overproduction caused the formation of ectopic growth zones and cell branching; thus, this protein was proposed to constitute a structural component of an organizing center for PG synthesis during polar growth (16). However, its exact function is yet to be determined.

In S. meliloti, 7TMR-DISM (transmembrane receptors with diverse intracellular signaling modules) cyclic di-GMP phosphodiesterase RgsP (Rgs for rhizobial growth and septation) and putative membrane-anchored periplasmic PG metallopeptidase RgsM were identified as important factors for unipolar cell growth (12). Both are essential proteins and conserved in *Rhizobiales* (12). These proteins localize to sites of zonal PG synthesis at the growing cell pole and the septum. Depletion of RgsP or RgsM results in cell growth inhibition and altered muropeptide composition (12).

Here, we expand the knowledge on the components involved in the control of unipolar cell growth and division in *Rhizobiales*. We report nine further S. meliloti proteins with unknown functions that localize to sites of zonal PG synthesis, are required for cell growth, and are involved in protein-protein interactions with RgsP and RgsM. We further show that Rgs proteins interact with components of the Tol-Pal system, which is localized to sites of zonal PG synthesis and essential for S. meliloti cell division.

## RESULTS

### RgsA is essential and localizes to sites of zonal cell wall growth.

**FIG 1:**
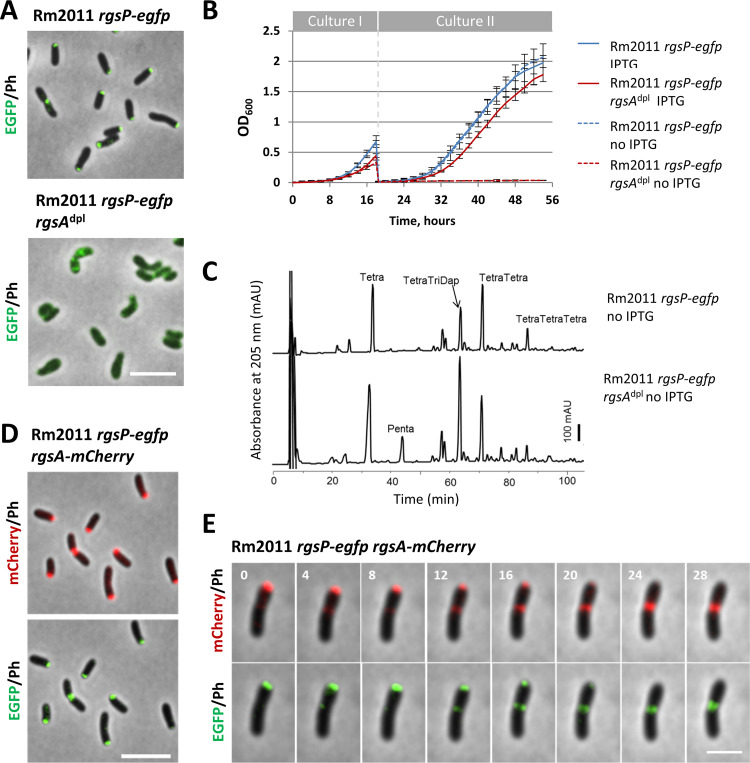
Similarly to its interaction partner RgsP, RgsA is essential for growth and normal muropeptide composition and localizes to the growing cell pole and septum. (A) Fluorescence microscopy images of Rm2011 *rgsP-egfp* and its *rgsA* depletion derivative. Cells from TY cultures, grown for 20 h without added IPTG, are shown. Ph, phase contrast. Bar, 5 μm. (B) Growth of Rm2011 *rgsP-egfp* and its *rgsA* depletion derivative. Culture I samples were inoculated at an OD_600_ of 0.005 in TY medium either with or without added IPTG, grown for 18 h, and set back to an OD_600_ of 0.005 in fresh TY medium with or without added IPTG to start culture II. (C) Muropeptide profiles of PG samples from Rm2011 *rgsP-egfp* and its *rgsA* depletion derivative, grown for 20 h in TY medium without IPTG. (D) Fluorescence microscopy images of Rm2011 *rgsP-egfp rgsA-mCherry*. Cells from exponentially growing TY cultures are shown. Bar, 5 μm. (E) Time-lapse microscopy images of Rm2011 *rgsP-egfp rgsA-mCherry*. Time is denoted in minutes. Ph, phase contrast. Bar, 2 μm.

We previously identified RgsA (SMc00644) as a potential interaction partner of RgsP and RgsM (12), and recently, a massively parallel transposon insertion sequencing (Tn-seq) study suggested a large growth impairment is caused by transposon insertions in *rgsA* (17). To test for a functional relation between RgsA, RgsP, and RgsM, we attempted to knock out *rgsA* in strain Rm2011 *rgsP-egfp*, carrying the gene fusion in place of *rgsP* at the native genomic locus. Since this approach failed, we constructed the RgsA depletion strain Rm2011 *rgsP-egfp rgsA*^dpl^. To enable RgsA depletion in Rm2011 *rgsP-egfp*, the chromosomal *rgsA* gene was deleted in the presence of a plasmid-borne copy of this gene controlled by its native promoter. Replication of this single copy plasmid pGCH14 was mediated by a *repABC* operon whose expression was dependent on isopropyl-β-d-thiogalactopyranoside (IPTG) (18). Omitting IPTG from the growth medium of this RgsA depletion strain resulted in loss of normal RgsP localization and growth inhibition (Fig. 1A and B). In contrast, with IPTG added to the medium, the RgsA depletion strain grew normally (Fig. 1B). RgsA-depleted cells were slightly shorter and wider than the wild-type cells and showed increased cell curvature (Fig. 1A, Table 1). This cell morphology was different from the shorter rounded rods observed upon RgsP or RgsM depletion (12). Muropeptide analysis of PGs isolated from RgsA-depleted cells revealed the following changes in the PG composition: increases in monomeric tetra- and pentapeptides and the glycine-containing dimer [TetraTetra(Gly4)], and a decrease in the dimeric (TetraTetra) and trimeric (TetraTetraTetra) cross-linked muropeptides (Fig. 1C, Table 2). These changes in muropeptide composition are similar to those in RgsP- or RgsM-depleted cells reported previously (12). To monitor cellular localization of RgsA by fluorescence microscopy, we replaced *rgsA* with *rgsA-mCherry* at the native genomic location in the *rgsP-egfp* strain. The resulting strain did not differ from the wild type in growth and cell morphology; thus, we concluded that the protein fusion was functional. RgsA-mCherry colocalized with RgsP-enhanced green fluorescent protein (EGFP) at the growing cell pole and the septum (Fig. 1D). RgsA-mCherry and RgsP-EGFP fluorescent foci tracked by time-lapse microscopy showed matching localization dynamics during the cell cycle and during their relocation from the growing pole to the septum (Fig. 1E; see also Fig. S2 in the supplemental material), corroborating the suggested close functional relation between the two proteins. These results support the notion that RgsA may constitute a part of a protein complex including RgsP and RgsM required for S. meliloti cell wall growth.

**TABLE 1:** Mean values of cell morphology measurements in cultures, grown without added IPTG for 20–24 h

Strain	Length	Width	Area	Curvature	Roundness	No.^*a*^
2011 *rgsP-egfp*	3.15	0.78	2.26	0.14	0.30	918
2011 *rgsP-egfp rgsA*^dpl^	2.51	0.83	1.87	0.32	0.42	1,000
2011 *rgsP-egfp rgsB*^dpl^	2.64	0.85	2.00	0.20	0.39	1,000
2011 *rgsP-egfp rgsC*^dpl^	3.00	0.88	2.44	0.18	0.36	798
2011 *rgsP-egfp rgsD*^dpl^	2.16	0.95	1.77	0.21	0.50	583
2011 *rgsP-egfp rgsE*^dpl^	2.27	0.93	2.12	0.77	0.55	1,000
2011 *rgsP-egfp rgsF*^dpl^	2.07	0.89	1.64	0.25	0.51	1,000
2011 *rgsP-egfp rgsG*^dpl^	2.64	0.83	2.01	0.19	0.39	813
2011 *rgsP-egfp rgsH*^dpl^	2.63	0.80	1.90	0.18	0.38	1,000
2011 *rgsP-egfp tolQ*^dpl^	3.64	0.77	2.63	0.19	0.29	601
2011 *rgsP-egfp pal*^dpl^	3.49	0.77	2.49	0.17	0.29	1,000

a Number of analyzed cells.

**TABLE 2:** Relative amount (%) of muropeptides in PG samples

Muropeptide	Relative amount (%) in strain^*a*^:	
	2011 *rgsP-egfp*	2011 *rgsP-egfp rgsA*^dpl^
Tri	3.0 ± 1.2	2.6 ± 0.0
TetraGly4	4.5 ± 0.2	3.7 ± 0.0
Tetra	24.5 ± 0.6	32.9 ± 0.3
Penta	ND^*b*^	7.6 ± 0.6
TetraTriDapGly4	6.8 ± 0.2	5.3 ± 0.2
TriTriDap	3.4 ± 0.4	3.3 ± 0.1
TetraTetraGly4	15.5 ± 0.1	22.7 ± 0.7
TetraTriDap	3.5 ± 0.1	2.0 ± 0.1
TetraTri	1.0 ± 0.1	1.0 ± 0.1
TetraTetra	24.0 ± 0.3	11.8 ± 0.8
TetraAnh	1.5 ± 0.0	1.1 ± 0.1
TetraTetraTriDap	2.7 ± 0.6	2.5 ± 0.2
PentaAnh	2.3 ± 0.0	1.0 ± 0.0
TetraTetraTetra	7.4 ± 0.2	2.5 ± 0.2

a Values are mean ± variation of two biological repeats.

b ND, not detected.

### RgsA, RgsM, RgsP, several hypothetical proteins, and components of the Tol-Pal system constitute an interaction network.

To test the hypothesis of a functional connection between RgsP, RgsM, RgsA, and known cell wall growth factors, we screened for additional protein interaction partners. For this purpose, we replaced the native *rgsA* by a 3×FLAG-tagged gene version to generate strain Rm2011 *rgsP-egfp rgsA-*3×*flag*. Exponentially growing cells of this strain were subjected to cross-linking with formaldehyde followed by application of cell lysates to an α-FLAG affinity matrix and mass spectrometry-assisted protein identification of the immunopurified samples. A control strain carrying the nontagged wild-type *rgsA* was analyzed in parallel to determine nonspecific protein enrichment in the pulldown assay used.

**FIG 2:**
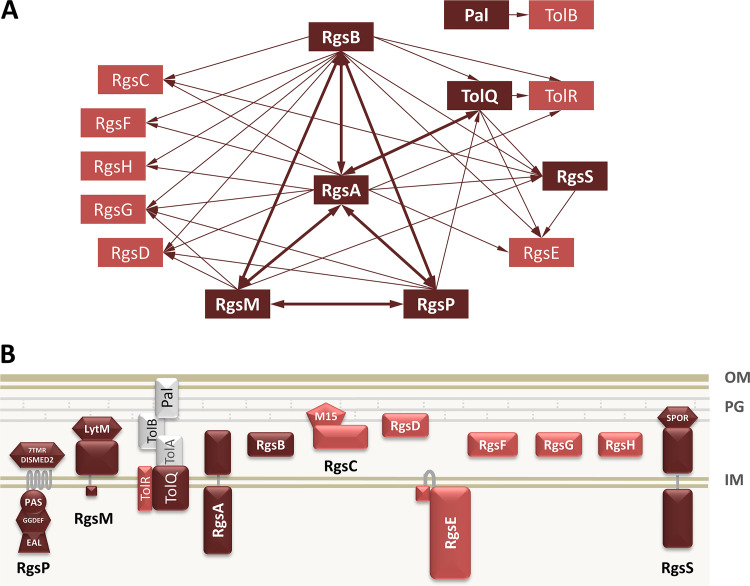
Identification of novel polar growth factors in S. meliloti. (A) Summary of putative protein-protein interactions identified in pulldown experiments. Dark red rectangles indicate proteins used as baits. Thick arrows indicate reciprocal pulldown. (B) Schematic representation of the membrane topology and conserved domains in Rgs proteins and the Tol-Pal system.

RgsP and RgsM were enriched in the RgsA pulldown sample, implying that these three proteins may form a complex *in vivo* (Fig. 2A; see Data Set S1). Moreover, among the RgsA-coimmunoprecipitated proteins, we found the TolQ and TolR components of the Tol-Pal system and eight hypothetical proteins encoded by genes of unknown function assigned to the category “essential” or “large growth impairment” in the recent Tn-seq study (17) (Data Set S1). These proteins were named RgsB (SMc04006), RgsC (SMc04010), RgsD (SMc01011), RgsE (SMc00190), RgsF (SMc03995), RgsG (SMc00950), RgsH (SMc00153), and RgsS (SMc02072) (Fig. 2A). RgsE is a homolog of the A. tumefaciens growth pole ring protein GPR, recently reported to promote polar growth in this organism (16). Noteworthy, RgsB, RgsD, and RgsG were previously identified as putative interaction partners of RgsM, and RgsG was identified as a putative interaction partner of RgsP (12).

10.1128/mBio.00306-20.6 DATA SET S1 Proteins identified in pulldown experiments as putative interaction partners of the indicated proteins. Relative enrichment (cutoff of 0.5 for experiment sample) was calculated as a fraction of the total signal in percent multiplied by the coverage value and used for the ranking. The essentiality index values are taken from diCenzo et al. (17). Essential genes have values close to zero. Blue highlights proteins analyzed in this study. Download Data Set S1, XLSX file, 0.1 MB. Copyright © 2020 Krol et al. 2020 Krol et al. This content is distributed under the terms of the Creative Commons Attribution 4.0 International license.

To corroborate the identified putative interactions, strains carrying *rgsB-*3×*flag*, *rgsS-*3×*flag* and *tolQ-*3×*flag* were used in pulldown assays. Rgs proteins identified in the RgsB-3×FLAG and RgsA-3×FLAG pulldown samples largely overlapped (Fig. 2A; Data Set S1). Moreover, RgsB was the most strongly enriched coimmunoprecipitated protein in the RgsA-3xFLAG sample and *vice versa*. In the RgsS-3×FLAG pulldown sample, RgsE and RgsC were among the enriched proteins, and pulldown with TolQ-3×FLAG identified TolR, TolA, RgsA, RgsS, and RgsE as enriched. Moreover, we used strains generated previously to repeat the pulldown experiments with RgsP-3×FLAG and RgsM-3×FLAG (12). The resulting data corroborated our previous findings and added links between the RgsP-RgsM interaction network and TolQ and RgsS (Fig. 2A; Data Set S1). Collectively, our coimmunoprecipitation results suggest protein-protein interactions between Rgs proteins and the Tol-Pal system.

We did not find the outer membrane-anchored and periplasmic components of the Tol-Pal system, TolB and Pal, among the interaction partners of TolQ or Rgs proteins. Thus, we carried out a pulldown experiment with a strain carrying *pal-*3×*flag* at the native genomic location. In this assay, only TolB was strongly enriched, indicating that Pal is unlikely to directly interact with Rgs proteins and inner membrane components of the Tol-Pal system (Fig. 2A; Data Set S1).

### Membrane topology of novel Rgs proteins and their interactions in a bacterial two-hybrid assay.

To get first hints on properties of the novel Rgs proteins, we analyzed their protein sequences with the Phobius transmembrane topology prediction tool (19). In RgsA and RgsS, a large N-terminal cytoplasmic domain and a C-terminal periplasmic domain separated by a transmembrane helix were predicted. RgsE was predicted to be primarily located in the cytoplasm, anchored in the inner membrane via two transmembrane helices (Fig. 2B; Data Set S2). Periplasmic location and signal peptides were predicted for RgsB, RgsD, RgsG, RgsH, and RgsF, whereas RgsC was suggested to be an extracytoplasmic protein without a signal peptide (Fig. 2B; Data Set S2).

10.1128/mBio.00306-20.7 DATA SET S2 Features of Rgs proteins deduced from analysis of their amino acid sequences. Asterisk indicates that the analyzed protein sequence was N-terminally extended with 43 amino acids. Download Data Set S2, XLSX file, 0.1 MB. Copyright © 2020 Krol et al. 2020 Krol et al. This content is distributed under the terms of the Creative Commons Attribution 4.0 International license.

To validate the predicted membrane topology, we used fusions to the partial E. coli alkaline phosphatase PhoA_27–471_, lacking the signal peptide. PhoA is only enzymatically active if located in the periplasm, where its activity results in blue staining of the bacterial culture on indicator plates. Rgs protein-coding sequences of different lengths were inserted into a plasmid carrying the PhoA_27–471_ coding sequence, and the resulting plasmids were introduced into S. meliloti Rm2011 and E. coli S17-1. Periplasmic location, dependent on the signal peptide, was confirmed for RgsB, RgsD, RgsF, RgsG, and RgsH, since fusions of the full-length proteins to PhoA_27–471_ produced blue staining of the respective bacterial cultures in both species and fusions of proteins lacking the predicted signal peptide portion did not produce blue staining (Fig. S1A). Furthermore, cytoplasmic location of the N-terminal portion, periplasmic location of the C-terminal portion, and location of the transmembrane segment in RgsA and RgsS were verified as well as the cytoplasmic location of RgsE and its membrane anchoring (Fig. S1A).

10.1128/mBio.00306-20.1 FIG S1 Experimental verification of Rgs protein membrane topology using fusions to E. coli PhoA2_7–471_. (A) Rgs-PhoA_27–471_ protein fusions of different lengths were analyzed for periplasmic location of PhoA. (B) Validation of the N-terminally extended RgsC protein sequence, containing the signal peptide. Partial amino acid sequence of RgsC and regions fused to PhoA_27–471_ are shown. Location in the periplasm was indicated by the ability of the strain that carried the fusion to hydrolyze the PhoA substrate, resulting in blue staining. Respective coding sequences were inserted into the plasmid pSRKKm-phoA (in case of RgsC1-605, *rgsC-phoA* was inserted into pWBT), and resulting constructs were introduced into S. meliloti Rm2011 (*Sm*) or E. coli S17-1 (*Ec*). EV, empty vector pSRKKm-phoA. Ten microliters of cell suspension was spotted onto the agar plates containing PhoA substrate and grown for 24 h. Download FIG S1, TIF file, 2.7 MB. Copyright © 2020 Krol et al. 2020 Krol et al. This content is distributed under the terms of the Creative Commons Attribution 4.0 International license.

10.1128/mBio.00306-20.2 FIG S2 Growth in liquid culture and fluorescence time-lapse microscopy analysis of Rm2011 *rgsP-egfp* carrying corresponding gene fusions at the native genomic location. (A) Growth of Rm2011 *rgsP*-*egfp* and its derivatives, carrying the indicated fluorescence protein gene fusions in TY medium. Cultures were inoculated at an OD_600_ of 0.005. Error bars represent standard deviations from three biological replicates. (B to L) Time-lapse fluorescence microscopy. Time is denoted in minutes. Ph, phase contrast. Bar, 2 μm. (B) *rgsA-mCherry*. (C) *rgsB-mCherry*. (D) *rgsC-mCherry*. (E) *rgsD-mCherry*. (F) *rgsE-mCherry*. (G) *rgsF-mCherry*. (H) *rgsG*-*mCherry*, (I) *rgsH-mCherry*. (K) *mVenus-rgsS*. (K) *tolQ-mCherry*. (L) *pal-mCherry*. Download FIG S2, JPG file, 2.3 MB. Copyright © 2020 Krol et al. 2020 Krol et al. This content is distributed under the terms of the Creative Commons Attribution 4.0 International license.

Comparison of the annotated RgsC sequence with homologous sequences in databases suggested a longer protein, with an additional 43 amino acids at the N terminus (Fig. S1B). This increased the length of the protein to 605 amino acids. RgsC_1–605_ fused to PhoA_27–471_ produced blue staining on indicator plates, which suggested periplasmic location of the fusion protein. Thus, we used fusions to PhoA to determine the length of the signal peptide. Fusion of the first 35 amino acids of RgsC to PhoA_27–471_ did not result in blue staining in strains bearing the corresponding construct, whereas a similar construct carrying the first 38 amino acids produced the staining (Fig. S1). This indicates that the signal peptide of RgsC is 36 to 38 amino acids long. The cysteine at position 37 might represent an acylation site, typically located directly after the signal peptide cleavage site in lipoproteins (20).

**FIG 3:**
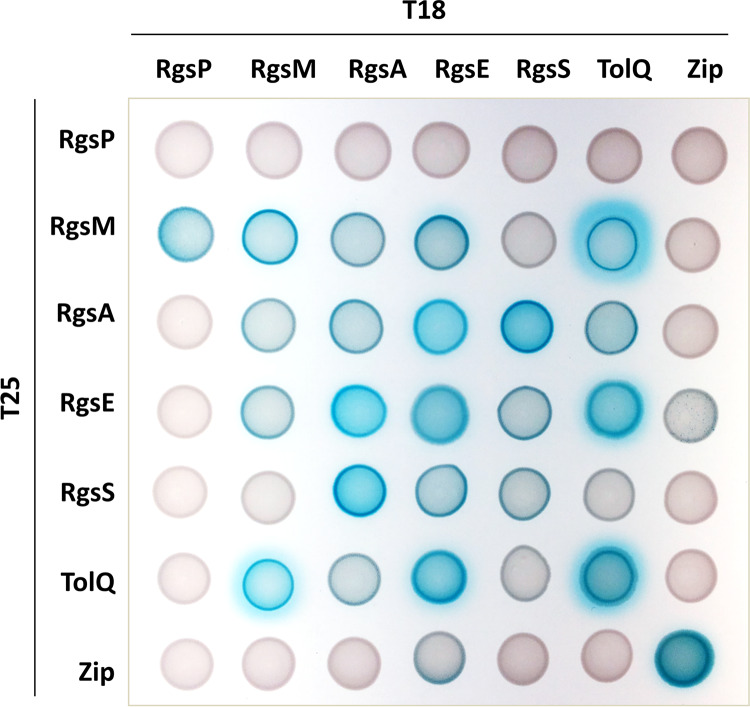
Bacterial two-hybrid analysis. E. coli strain BTH101 was cotransformed with the indicated T18 and T25 fusion constructs, and 10 μl of the cotransformant cell suspensions was spotted onto LB agar containing X-Gal. Blue staining indicates protein-protein interactions.

Protein-protein interactions were tested in a bacterial two-hybrid system. We employed a system based on reconstitution of active adenylate cyclase from T18 and T25 fragments upon interaction of the fused proteins of interest. β-Galactosidase activity as the signal output produces blue staining on indicator plates with X-Gal (5-bromo-4-chloro-3-indolyl-β-d-galactopyranoside). Since adenylate cyclase is a cytoplasmic enzyme, we only fused the T18 and T25 fragments to proteins containing cytoplasmic domains. Strong blue staining indicated interaction of TolQ with RgsM, RgsE, and itself as well as of RgsA with RgsE and RgsS. Weaker staining was caused by combining RgsM with RgsP, RgsA with itself, RgsA with TolQ and itself, and RgsE with RgsS (Fig. 3). The negative result obtained by combining RgsA with RgsP might be a hint that the RgsP-RgsA interaction is indirect, possibly mediated by RgsM. Alternatively, it might be due to unfavorable conformations of RgsA and RgsP proteins fused to the adenylate cyclase fragments.

### Rgs proteins and Tol-Pal are required for growth of S. meliloti.

**FIG 4:**
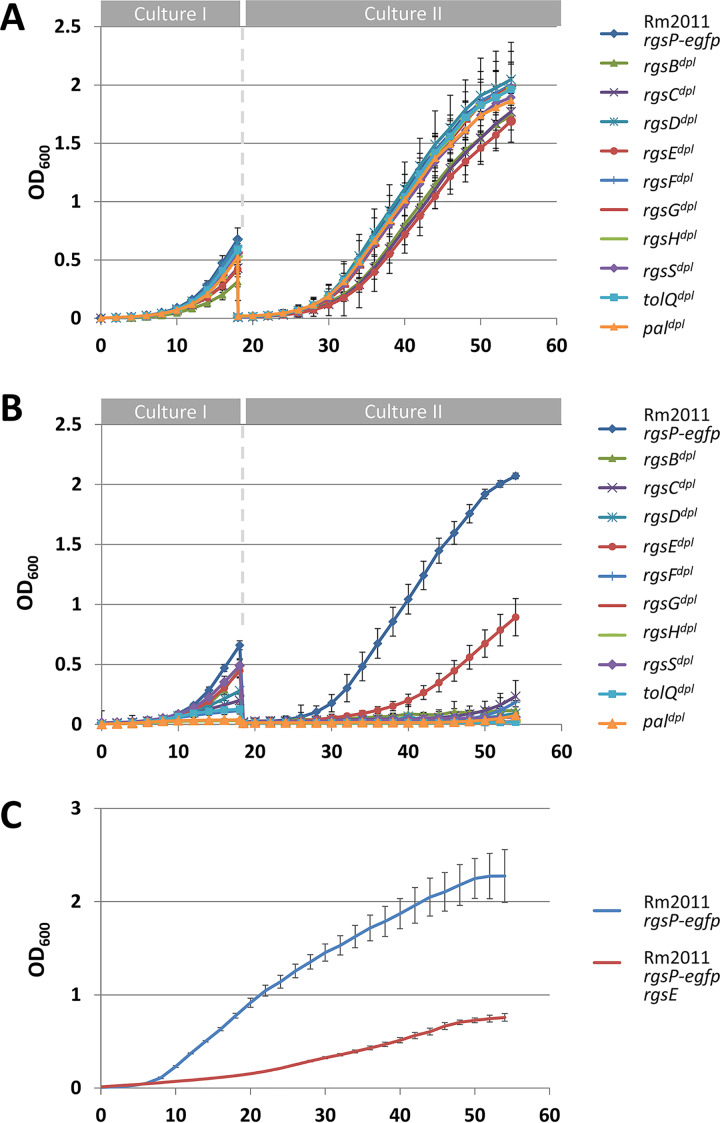
Effects of TolQ, Pal, and novel Rgs protein depletion on growth. (A and B) Growth of Rm2011 *rgsP-egfp* (control) and its *rgs* or *tol-pal* depletion derivatives in TY medium. Culture I samples either with added IPTG (A) or without added IPTG (B) were inoculated at an OD_600_ of 0.005, grown for 18 h, and used to start culture II samples in fresh TY medium with or without added IPTG at an OD_600_ of 0.005. Error bars indicate standard deviations from three biological replicates. (C) Growth of Rm2011 *rgsP-egfp rgsE* strain and its parental strain Rm2011 *rgsP-egfp* in TY medium. Cultures were inoculated at an OD_600_ of 0.01. Error bars indicate standard deviations from six biological replicates.

Our attempts to generate knockout mutants failed for all the novel *rgs* genes, *tolQ*, and *pal*. Thus, we constructed depletion strains in the Rm2011 *rgsP-egfp* genetic background. To generate RgsB, RgsC, RgsD, RgsE, RgsF, RgsG, RgsH, RgsS, TolQ, and Pal depletion strains, the encoding gene was either placed under the control of an IPTG-inducible promoter or placed under the control of the native promoter on a single-copy plasmid whose replication is IPTG dependent (see Materials and Methods). Growth of the depletion strains in medium with added IPTG was similar to that of the wild type (Fig. 4A), whereas without added IPTG, growth was inhibited (Fig. 4B). The onset of growth at the end of the cultivation without added IPTG, observed for several strains, can likely be attributed to occurrence of suppressor mutants able to overcome the LacI-mediated transcription repression. RgsE depletion resulted in slow but constant growth, suggesting that cell proliferation is possible in the absence of RgsE. After plating the Rm2011 *rgsP-egfp rgsE^dpl^* strain on tryptone-yeast extract (TY) agar without added IPTG, we were able to isolate single colonies of the Rm2011 *rgsP-egfp rgsE* strain, which lost the curable complementation plasmid pGCH14-rgsE. Growth of this strain was similar to that of Rm2011 *rgsP-egfp rgsE^dpl^* cultivated without IPTG (Fig. 4C).

**FIG 5:**
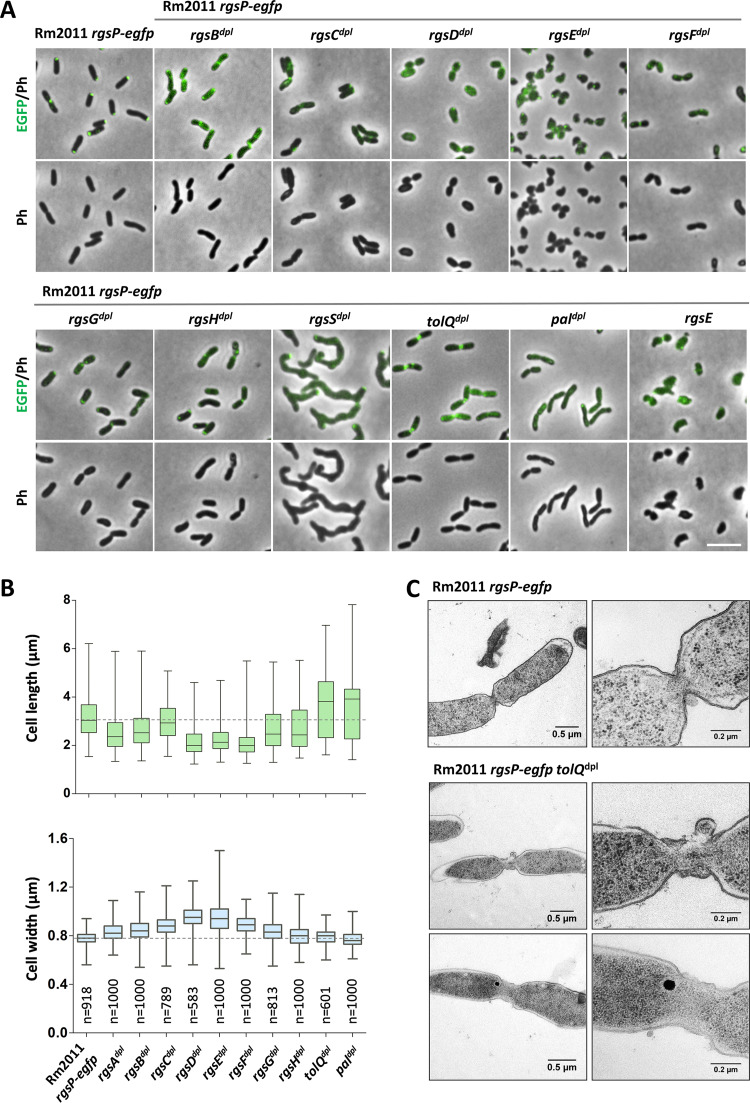
Effects of TolQ, Pal, and novel Rgs protein depletion on cell morphology and RgsP localization. (A) Fluorescence microscopy images of Rm2011 *rgsP-egfp*, and its *rgs* or *tol-pal* depletion derivatives, grown in TY without added IPTG for 20 to 24 h. Ph, phase contrast. Bar, 5 μm. (B) Distribution of cell lengths and widths in cultures, grown in TY without added IPTG for 20 to 24 h. The boxes show 25th and 75th percentiles separated by the lane indicating the median value, and whiskers show minimum and maximum values. Numbers of analyzed cells are shown in the lower panel. The dashed line shows the median value of strain Rm2011 *rgsP-egfp.* (C) Transmission electron microscopy images of Rm2011 *rgsP-egfp* and its *tolQ* depletion derivative grown in TY without added IPTG for 20 h.

In cells depleted for Rgs or Tol-Pal proteins, the characteristic polar and septal RgsP-EGFP localization was either diminished or lost (Fig. 5A). Moreover, Rgs and Tol-Pal depletion resulted in significant changes in cell length and width as well as alterations in cell area, roundness, and curvature (Fig. 5A and B, Table 1). Whereas almost normal rod cell morphology was retained under RgsB, RgsC, RgsG, and RgsH depletion conditions (Fig. 5A and B), cells depleted for RgsD or RgsF were strongly shortened and rounded, similar to RgsP- and RgsM-depleted cells (12). RgsE-depleted cells were irregularly shaped with strong curvature. These cells were morphologically similar to the A. tumefaciens GPR-depleted cells (16). Depletion of RgsS resulted in the formation of short partially branched cell filaments with bulges along the filaments, which may represent swollen septation sites. This implies that RgsS is indispensable for cell division. However, since the filament tips showed RgsP-EGFP-derived fluorescence signal and the cells were able to elongate to form short filaments, RgsS is probably not strictly required for cell elongation (Fig. 5A). Upon depletion of TolQ or Pal, a strong increase in the mean cell length accompanied by minor changes in cell width was reflective of an accumulation of predivisional doublets of normally rod-shaped cells, separated by constricted septa (Fig. 5B). This implies that the Tol-Pal system is not crucial for generation of normal S. meliloti rod cell shape but is required for completion of cell division. Electron microscopy of TolQ-depleted cells revealed extended tube-like constricted septa, corroborating a defect in late stages of cell division (Fig. 5C).

### Novel Rgs proteins and Tol-Pal localize to cell wall growth zones.

To determine the subcellular localization of the Tol-Pal system and the newly identified Rgs proteins, we replaced the *pal* and *rgs* genes at their native genomic locations with versions of these genes encoding fusions to fluorescent proteins. The Rm2011 *rgsP-egfp tolQ-mCherry* strain was generated by insertion of the gene fusion as a single transcription unit driven by a copy of the *tolQ* promoter immediately upstream of the *tolQRAB* operon. All the resulting strains, except for Rm2011 *rgsP-egfp rgsC-mCherry*, grew at rates indistinguishable from that of the wild type (Fig. S2A), strongly suggesting that the produced protein fusions were functional. The strain producing RgsC-mCherry showed a slight slowdown of growth in the exponential phase, indicative of a minor loss of RgsC functionality due to the fusion to mCherry (Fig. S2A).

**FIG 6:**
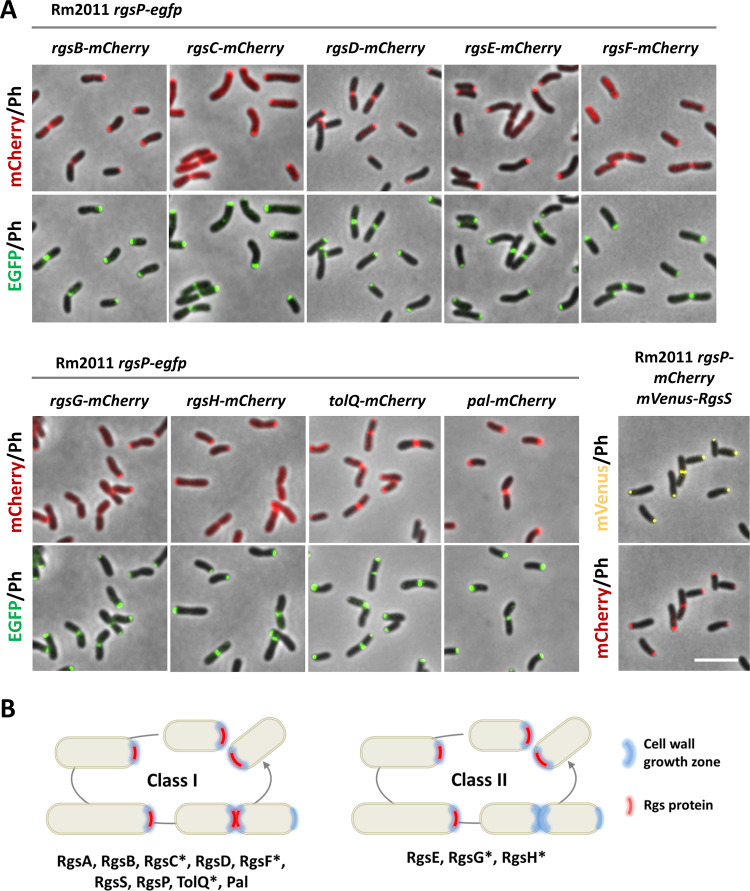
Colocalization of novel Rgs proteins, TolQ, and Pal with RgsP-EGFP. (A) Fluorescence microscopy images of Rm2011 *rgsP-egfp* carrying *mCherry* inserted at the native genomic location of *rgsG*, *rgsH*, *tolQ*, and *pal* to generate C-terminal mCherry fusions to the respective proteins, and Rm2011 *rgsP-mCherry* carrying *mVenus-rgsS* at the native genomic location, growing exponentially in TY broth. Bar, 5 μm. (B) Summary of the time-lapse fluorescence microscopy analysis shown in Fig. S2 in the supplemental material. Class I Rgs proteins are localized at the growing pole and septum. Class II Rgs proteins are localized at the growing pole only. Asterisk indicates protein fusions that produced diffuse fluorescence signal in addition to localized foci.

Microscopy analysis of these strains in the exponential growth phase revealed polar fluorescent foci, which colocalized with RgsP-EGFP or RgsP-mCherry foci. RgsC-, RgsF-, RgsG-, and TolQ-mCherry additionally generated diffuse fluorescent signals (Fig. 6A). We cannot exclude that this spatially diffuse signal distribution was caused completely or partially by proteolytic release of mCherry from the fusion proteins and that Rgs proteins released from these fusion proteins may have contributed to the overall activity of the respective Rgs proteins. Septal foci of fluorescence were detected for RgsB-, RgsC-, RgsD-, RgsF-, TolQ-, and Pal-mCherry as well as for mVenus-RgsS (Fig. 6A). Colocalization of the Tol-Pal system and the newly identified Rgs proteins with RgsP imply that they accumulate in the areas of cell wall biosynthesis.

The results of the microscopy of exponential culture samples were confirmed in time-lapse studies. RgsB-, RgsC-, RgsD-, RgsF-, TolQ-, and Pal-mCherry colocalized with RgsP-EGFP, as well as mVenus-RgsS with RgsP-mCherry, over the whole cell cycle. In contrast, RgsE-, RgsG-, and RgsH-mCherry formed fluorescent foci at the growing pole and not at the septum (Fig. S2). In this time-lapse analysis, transient accumulation of spatially diffuse RgsG-mCherry signal in the nongrowing cell compartment during a short period before cell constriction was observed in most of the cells analyzed (Fig. S2H).

Taken together, these observations suggest that Rgs proteins and the Tol-Pal system are spatiotemporally coordinated. We grouped the Rgs proteins according to their localization pattern. Class I Rgs proteins and Tol-Pal proteins were associated with sites of zonal cell wall biosynthesis during cell elongation and cell division, whereas class II Rgs proteins were only localized at the site of polar cell elongation (Fig. 6B).

### Domain structure and conservation of novel Rgs proteins.

**FIG 7:**
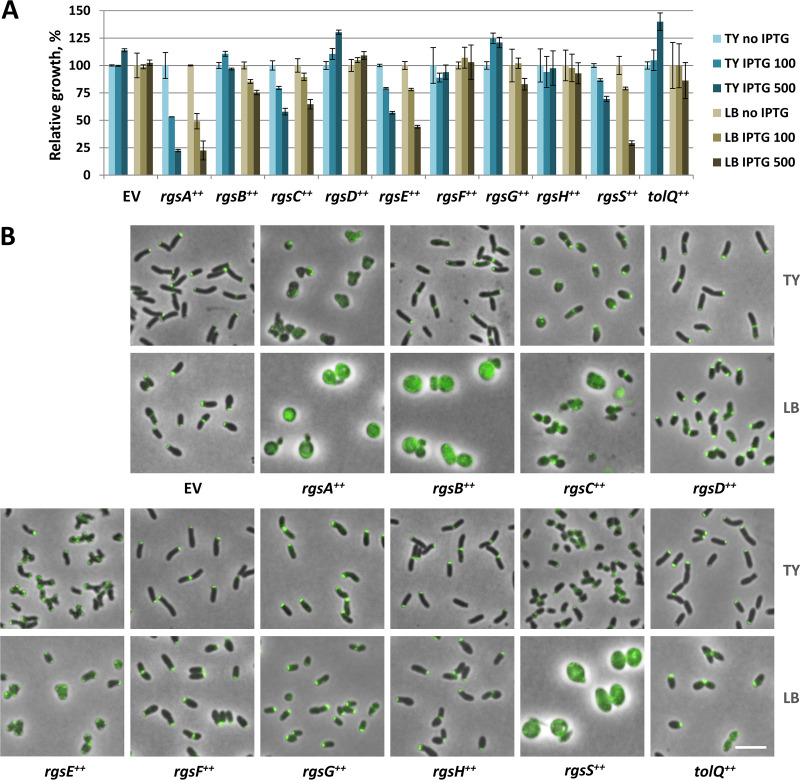
Effects of *rgs* and *tolQ* gene overexpression on cell morphology and growth. (A) Relative growth of Rm2011 *rgsP-egfp* carrying the empty vector pWBT (EV) or *rgs* and *tolQ* overexpression constructs. Precultures grown in TY medium without IPTG were used to inoculate TY or LB cultures with IPTG added at 0, 100, or 500 μm. The optical density was measured after 16 h of growth. Values normalized to the growth of the cultures without added IPTG are shown. Error bars indicate standard deviations from three biological replicates. (B) Fluorescence microscopy images of Rm2011 *rgsP-egfp* cells, carrying the empty vector pWBT (EV) or the indicated gene overexpression constructs (*gene*^++^), grown in TY or LB broth with added 500 μm IPTG for 20 to 24 h. Merged phase contrast and EGFP fluorescence images are shown. Bar, 5 μm.

Since all novel Rgs proteins were annotated as hypothetical, we applied computational prediction tools to search for conserved domains and characteristic structural features providing first hints at their possible functions (Fig. 2B). Furthermore, we overexpressed the corresponding genes in Rm2011 *rgsP-egfp* and analyzed growth and cell morphology. For this analysis, we used complex TY and LB media. TY medium, containing 2.5 mM CaCl_2_, is routinely used for the cultivation of S. meliloti, whereas LB medium does not contain CaCl_2_ and was observed previously to potentiate the effect of cell surface-related defects on S. meliloti cell morphology (12). Homology and structural similarity analyses of RgsA, RgsB, and RgsH did not provide any hint at their possible functions, whereas RgsG contained the conserved domain IalB (COG5342, invasion-associated locus B) (Data Sets S2 and S3). Overexpression of *rgsA* in Rm2011 *rgsP-egfp* strongly inhibited growth in both media and caused delocalization of RgsP-EGFP and loss of the rod shape (Fig. 7). In LB broth, *rgsA*-overexpressing cells were strongly enlarged. Overexpression of *rgsB* in TY-cultivated cells had no effect on cell growth and morphology or on localization of RgsP-EGFP. In cells cultured in LB, *rgsB* overexpression resulted in a phenotype similar to that caused by *rgsA* overexpression. Enhanced expression of *rgsG* and *rgsH* had no effect in either media (Fig. 7).

RgsC contains a conserved M15 Zn metallopeptidase domain in its N-terminal portion (Data Set S2). Structure modeling of RgsC using the SWISS-MODEL online modeling tool (21) identified structural similarities to dd- and ld-carboxypeptidases from Gram-positive bacteria and the l-Ala-d-Glu endopeptidase domain of bacteriophage endolysin within the M15 peptidase domain (Data Set S3). Overexpression of *rgsC* in TY-grown cells moderately affected growth and resulted in coccoid cells partially retaining proper RgsP-EGFP localization. In LB broth, the *rgsC* overexpression phenotype included cell lysis (Fig. 7).

10.1128/mBio.00306-20.8 DATA SET S3 Similarities of Rgs proteins to proteins with known crystal structure, deduced from analysis of amino acid sequences using SWISS-MODEL online tool. Download Data Set S3, XLSX file, 0.1 MB. Copyright © 2020 Krol et al. 2020 Krol et al. This content is distributed under the terms of the Creative Commons Attribution 4.0 International license.

RgsE is a 2,089-amino-acid protein with up to ten apolipoprotein A1/A4/E domains, homology to SMC chromosome segregation protein (coiled-coil regions), and 42% sequence identity to A. tumefaciens GPR (Atu1348) (16). Overexpression of *rgsE* in cells cultured in TY broth resulted in cell branching (Fig. 7), similar to the morphology of A. tumefaciens GPR-overproducing cells (16). At the cell poles of the branches, polar foci of RgsP-EGFP mediated fluorescence were present. LB-grown *rgsE*-overexpressing cells were slightly enlarged and showed partially delocalized RgsP-EGFP signal (Fig. 7).

RgsF contains a conserved tetratricopeptide TPR domain and six Sel1-like repeats (Data Set S2), suggesting possible functions in protein-protein interactions and signal transduction. Overexpression of *rgsF* did not affect cell growth and morphology in either medium (Fig. 7).

The C-terminal portion of RgsS (residues 864 to 945) constitutes a conserved SPOR domain showing almost an equal degree of sequence similarity to the SPOR domains of E. coli FtsN and Bacillus subtilis cell wall amidase CwlC, whose crystal structures have been reported (22, 23) (see Fig. S3A and B; Data Set S2). Structure modeling revealed a characteristic arrangement of α-helices and β-strands in the SPOR domain of RgsS (Fig. S3C; Data Set S3). The remaining part of the RgsS amino acid sequence did not deliver significant homology hits pointing to its function. Overexpression of *rgsS* in cells cultured in TY broth resulted in slightly attenuated growth, shorter rods, and faint polar RgsP-EGFP foci, whereas in LB medium, growth was strongly reduced and cells were strongly enlarged, displaying a diffuse RgsP-EGFP signal (Fig. 7).

10.1128/mBio.00306-20.3 FIG S3 Structural features of the RgsS SPOR domain. (A) Alignment of RgsS and Bacillus subtilis CwlC SPOR domain regions. (B) Alignment of RgsS and E. coli FtsN SPOR domain regions. (C) Structure model of the RgsS SPOR domain generated using SWISS-MODEL online tool with B. subtilis CwlC as a template. N and C termini of the model are indicated. Download FIG S3, TIF file, 1.2 MB. Copyright © 2020 Krol et al. 2020 Krol et al. This content is distributed under the terms of the Creative Commons Attribution 4.0 International license.

**FIG 8:**
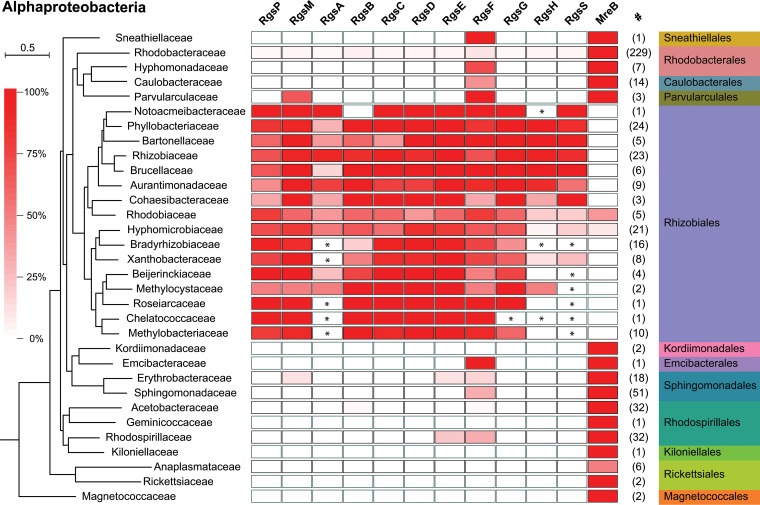
Conservation of novel Rgs proteins and MreB in *Alphaproteobacteria*. The color code indicates the proportion possessing a respective Rgs protein homolog, grouped at the family level. The number of considered complete proteomes in these groups is shown in column “#.” The phylogenetic tree was reconstructed using a maximum likelihood approach based on 121 single-copy orthologs common in all considered proteomes. The phylogenetic distance scale is shown at the top left. Asterisks indicate presence of at least one partial homolog, identified in a manual BLASTP search, in a given family.

Conservation of the Rgs proteins in alphaproteobacterial proteomes was analyzed by an iterative hidden Markov model (HMM)-assisted homology search. This revealed that RgsB, RgsC, RgsD, RgsE, and RgsF are well conserved in *Rhizobiales*, whereas RgsA, RgsH, and RgsS were only detected in alphaproteobacterial families phylogenetically close to *Rhizobiaceae* (Fig. 8; Data Set S5). We then employed a manual BLASTP search and identified shorter homologs sharing similarities with RgsA or RgsS in their C-terminal parts, constituting only a small portion of the cytoplasmic domain, transmembrane helix, and periplasmic domain (RgsA) or periplasmic domain (RgsS) (Fig. 8; Data Set S4). Full-length RgsH homologs were ultimately found in 13 of 16 considered *Rhizobiales* families (Fig. 8; Data Set S4). In alphaproteobacterial orders other than the *Rhizobiales*, only RgsE and RgsF homologs were found. In contrast, MreB homologs were found in the vast majority of *Alphaproteobacteria*, but in only four *Rhizobiales* species (Fig. 8). These were represented by two *Rhodobiaceae* and two *Hyphomicrobiaceae* species possessing MreB homologs but lacking Rgs homologs (Data Set S5). This suggests that presence of Rgs and MreB homologs is mutually exclusive. Rgs proteins were present in most representatives from the *Rhizobiaceae* and *Brucellaceae* and part of the *Hyphomicrobiaceae*, containing polarly growing species (11), which corroborates the assumption of their involvement in polar growth.

10.1128/mBio.00306-20.9 DATA SET S4 Putative Rgs homologs, indicated by “*” in Fig. 8, not identified in *Rhizobiales* by hidden Markov model-based iterative search due to partial or low homology. The proteins were identified using BLASTP. One representative homolog per phylum is shown. Download Data Set S4, XLSX file, 0.1 MB. Copyright © 2020 Krol et al. 2020 Krol et al. This content is distributed under the terms of the Creative Commons Attribution 4.0 International license.

10.1128/mBio.00306-20.10 DATA SET S5 Alignment of Rgs proteins in *Alphaproteobacteria*. Download Data Set S5, PDF file, 0.1 MB. Copyright © 2020 Krol et al. 2020 Krol et al. This content is distributed under the terms of the Creative Commons Attribution 4.0 International license.

### RgsD is a novel PG binding protein.

**FIG 9:**
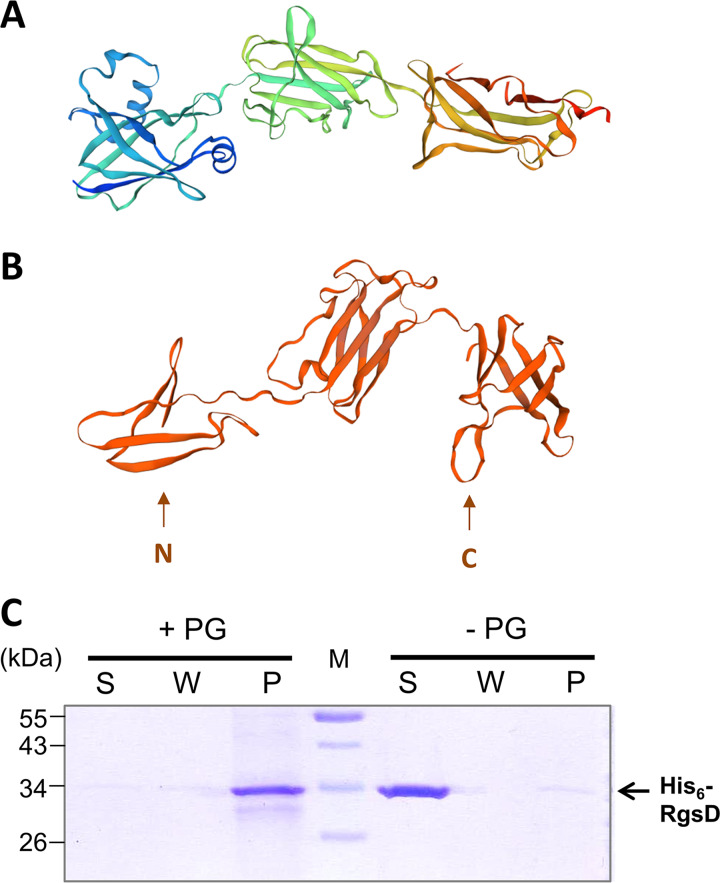
RgsD shows structural similarity to minor pilin RrgC and PG binding ability. (A) Ribbon diagram of experimentally determined RrgC structure (25). (B) Ribbon diagram of RgsD structure model determined by the SWISS-MODEL online tool with RrgC as a template. (C) PG binding ability of His_6_-RgsD_28–331_ was assessed in an *in vitro* binding assay with S. meliloti Rm2011 PG sacculi followed by SDS-PAGE and Coomassie blue staining. S, supernatant from the first centrifugation step; W, supernatant from the washing step; P, pellet; PG, peptidoglycan. Control reactions were performed in the absence of PG sacculi.

A homology search for RgsD did not identify any conserved domains; however, modeling the protein structure with SWISS-MODEL revealed structural similarities to isopeptide domains of thioester domain protein BaTIE from Bacillus anthracis and ancillary pilin RrgC from Streptococcus pneumoniae (Fig. 9A; Data Set S3). These are surface-associated proteins in Gram-positive bacteria and are suggested to participate in covalent adhesion. Whereas the target of BaTIE binding remains unknown (24), RrgC is covalently attached to the outer PG layer of the streptococcal cell wall (25). We reasoned that RgsD was likely to have affinity to PG and therefore tested whether it interacts with PG. Purified RgsD was retained in the pellet (P) sample following incubation with PG in a coprecipitation PG binding assay (Fig. 9B). Thus, RgsD likely represents a novel PG-binding protein.

## DISCUSSION

We previously identified RgsP and RgsM as novel proteins with unknown functions indispensable for S. meliloti growth and normal PG composition (12). Correlating with PG incorporation zones, these proteins were detected at the elongating cell pole and relocated to the septum prior to cell division. Here, we identified the Tol-Pal system and a further six proteins with yet-unknown functions, RgsA, RgsB, RgsC, RgsD, RgsF, and RgsS, which followed the spatiotemporal pattern of RgsP and RgsM and were required for S. meliloti growth. These represent a set of interacting proteins, associated with zones of cell wall synthesis during cell elongation and cell division, which we define as class I Rgs proteins. In contrast, RgsE, RgsG, and RgsH were only detected at the growing pole and not at the septum. These class II Rgs proteins might be specific to the polar cell elongation process.

### Class I Rgs proteins.

The periplasmic RgsD protein is exclusively conserved in Rhizobiales and was enriched in RgsA, RgsB, RgsP and RgsM pulldown samples. We identified RgsD as a novel PG-binding protein/domain. PG binding domains are often found in PG-processing enzymes (26). However, the RgsD amino acid sequence did not suggest any enzyme activity. Thus, RgsD could play a role in the positioning of PG synthesis factors or regulation of their activity.

Computational analysis of RgsA and RgsB sequences did not provide any hints to their functions. The sets of Rgs proteins enriched in RgsA and RgsB pulldown samples largely overlapped, suggesting that these two proteins might directly interact with each other. Depletion and overproduction phenotypes of RgsA were more pronounced than those of RgsB. Thus, RgsA possibly has a more important role for cell wall growth than RgsB, which might have an accessory function. Homologs of RgsB were found in all *Rhizobiales* families except for *Chelatococcaceae*, whereas RgsA appeared to be less conserved. Full-length RgsA homologs were mostly detected in families phylogenetically close to the *Rhizobiaceae*. In the remaining *Rhizobiales* families, shorter proteins with homology to the periplasmic portion of RgsA were present. We therefore speculate that the periplasmic RgsA portion might have a common role in all *Rhizobiales* families. In the RgsA and RgsB pulldown samples, TolQR and all the Rgs proteins considered in this work were enriched. This implies that RgsAB might be a core component in a Rgs-TolQR protein complex required for S. meliloti cell growth and division. This protein complex is likely dynamic, differing in composition between the polar cell elongation zone and the septum.

RgsC, present in all analyzed Rhizobiales families, contains a conserved M15 zinc-binding metallopeptidase domain and shows structural similarities to a dd-carboxypeptidase from Gram-positive Streptomyces albus G (27). dd-Carboxypeptidases remove the terminal d-alanine residue from pentamuropeptide in PG, thus regulating the abundance of substrates for transpeptidation by the major bifunctional PG synthases (28). In E. coli, eight dd-carboxypeptidases contribute to maintenance of the cell shape (29). S. meliloti possesses four annotated genes encoding putative dd-carboxypeptidases (*dac*, *SMc00996*, *SMc00683*, and *SMc00068*). These contain serine peptidase catalytic domains, characteristic for dd-carboxypeptidases in Gram-negative bacteria. Overexpression of *rgsC* resulted in rounded cells that were prone to lysis in medium without CaCl_2_, indicative of a cell envelope defect. Likewise, overexpression of *dacA* or *dacB* in E. coli increased cell envelope permeability (30). It remains to be determined if RgsC has PG carboxypeptidase activity.

RgsF represents the only Rgs protein that was substantially conserved outside *Rhizobiales*. It contains one tetratricopeptide (TRP) domain and six Sel1-like repeats. Domains of this kind mediate protein-protein interactions in all three kingdoms of life (31, 32). S. meliloti Sel1-like repeat protein ExoR regulates the ExoS-ChvI two-component regulatory system by binding to ExoS (33). Sel1-like repeat proteins were associated with virulence in pathogenic bacteria (34, 35). In Brucella abortus, periplasmic protein TtpA containing tetratricopeptide repeats (TPRs) is required for cell envelope integrity and virulence (36, 37). Interestingly, TPRs are present in E. coli LpoA, CpoB, and NlpI (38–40). LpoA is an outer membrane lipoprotein required for transpeptidase activity of PBP1A, one of the two major PG synthases in E. coli (41). The periplasmic protein CpoB regulates the LpoB-mediated transpeptidase stimulation of PBP1B dependent on the Tol energy state (39), and NlpI serves as adaptor protein for PG endopeptidases (40). RgsF does not share sequence homology with E. coli LpoA, CpoB, or NlpI; however, it is a promising candidate for a novel PG synthase activator in polarly growing *Rhizobiales*.

Of the six novel class I Rgs proteins, RgsS stood out, since its depletion resulted in cell filamentation, suggesting cell division arrest despite ongoing cell elongation. Similar to RgsA, full-length RgsS was found to be conserved only in families phylogenetically close to *Rhizobiaceae*. In the remaining *Rhizobiales* families, shorter RgsS homologs with similarities to the C-terminal part of RgsS containing a minor portion of the cytoplasmic domain, transmembrane helix, and periplasmic domain were found. RgsS is a transmembrane protein with conserved SPOR domain at the C terminus. These features are characteristic of FtsN from gammaproteobacterium E. coli and FtsN-like proteins from *Alpha*-, *Beta*-, and Deltaproteobacteria, which are highly variable at the sequence level (22, 42). Defects in FtsN or FtsN-like proteins resulted in cell filamentation due to cell division arrest (42, 43). Thus, RgsS may represent an FtsN-like protein. In E. coli, FtsN interacts with FtsZ-associated protein FtsA and PG biosynthesis-related proteins PBP1B, PBP3, FtsQ, and FtsW (44–46). Although S. meliloti possesses homologs of these proteins, they were not detected as enriched in the RgsS pulldown assay. In contrast to known FtsN-like proteins, whose cytoplasmic region is short (30 amino acids in FtsN-like CC2007 from Caulobacter crescentus), RgsS possesses a cytoplasmic domain of 600 amino acids. This suggests an additional or alternative functionality related to this cytoplasmic part. One such possible function might be related to cytoplasmic RgsS-RgsE interaction suggested by our pulldown and bacterial two-hybrid assays.

With accumulating knowledge on the genetics of polar cell wall growth, it became apparent that *Rhizobiales* might employ the cell division factors FtsZ, FtsA, and FtsW in the restrictive control of polar cell elongation by yet-unknown mechanisms (14, 15). Here, we show that in S. meliloti depletion of another putative cell division protein, the putative FtsN-like protein RgsS, resulted in cell filamentation and branching. Thus, we speculate that relocation of RgsS from the growing pole to the septum might be involved in cessation of polar growth.

### Class II Rgs proteins.

RgsE is a homolog of A. tumefaciens GPR, essential for growth pole formation in this bacterium (16). RgsE homologs were found in all *Rhizobiales* families and more distant homologs were detected in *Emcibacterales* and *Rhodospirillales*. However, no other Rgs protein homologs were detected in members of these orders. This raises the possibility of a different role for the RgsE homologs in spatiotemporal organization of these bacteria. RgsE and GPR are extraordinarily large membrane-anchored cytoplasmic proteins (2,089 and 2,115 amino acids, respectively), localized to the growing cell pole but absent from the septum. Depletion of RgsE introduced a strong cell morphology defect but did not completely abolish growth, and similarly to an A. tumefaciens strain lacking GPR (16), an *rgsE* deletion mutant was able to grow and divide, albeit aberrantly and slowly. Alterations in cell morphology upon depletion or overproduction of RgsE or GPR in their respective hosts are very similar, supporting a common function of these proteins. Interactions of RgsE with RgsA, RgsS, and TolQ, observed in this work, provide insights into the molecular environment of RgsE at the growing cell pole and thus suggest new directions for future studies of RgsE/GRP action in pole formation and polar growth.

Apart from RgsE, the class II Rgs proteins include the periplasmic proteins RgsG and RgsH. RgsG is well-conserved in *Rhizobiales* and contains an IalB domain. This domain was named after invasion-associated locus B from *Bartonella* species, which is an inner membrane protein, promoting the intracellular erythrocyte infection by this pathogen by an unknown mechanism (47). However, RgsG is not a direct homolog of IalB (BARBAKC583_0326) in Bartonella bacilliformis KC583, but a homolog of another similar protein, BARBAKC583_0800, in this species. RgsH was only partially conserved in *Rhizobiales*, and domains of known function could not be identified in this protein. The molecular functions of RgsG and RgsH responsible for their essentiality and localization to the growing pole remain unknown.

### Role of the Tol-Pal system.

Identification of TolQ and TolR among the putative interaction partners of RgsA, RgsB, and RgsP provides a link between Rgs proteins and known divisome components. Pulldown with TolQ as a bait enriched RgsA, RgsS, and RgsE, implying that these proteins might be closely associated with TolQ. The Tol-Pal system is dispensable in E. coli; however, it was reported to facilitate the cell division process via diverse mechanisms, such as maintaining cell envelope integrity, promoting the outer membrane constriction, regulating PBP1B transpeptidase activity, or indirectly activating amidases acting in septum splitting during cell division (39, 48–50). In the alphaproteobacterium Caulobacter crescentus, possessing MreB and performing dispersed cell elongation along the sidewall, the Tol-Pal complex is essential. It plays a role in maintaining the outer membrane integrity as well as in successful cell separation (51). Depletion of S. meliloti TolQ or Pal resulted in the accumulation of normally shaped predivisional cell doublets, suggesting that the final step in cell division was inhibited, but the remaining stages of the cell cycle proceeded normally. This phenotype was strikingly similar to that of TolA- or Pal-depleted C. crescentus, which showed doublets of fully developed normally shaped cells (51). Depletion of RgsP and RgsM also resulted in an accumulation of constricted predivisional cells, which indicates impaired cell division (12). It is tempting to speculate that RgsP, RgsM, and possibly further class I Rgs proteins might regulate Tol-Pal-associated processes at the final stages of S. meliloti cell division.

We show here that S. meliloti TolQ, Pal, and putative FtsN-like protein RgsS are localized at the growing cell pole and the septum. In C. crescentus, Tol-Pal proteins and FtsN-like CC2007 localized at the division plane and were retained at the new cell pole after cell division (42, 51). Absence of cell shape defects in the constricted doublets of TolQ- or Pal-depleted cells in both species suggests that these proteins are not required for maintaining a normal cell shape. Likewise, in both species, FtsN-like proteins are probably not strictly required for cell elongation, considering cell filamentation in their absence. In C. crescentus and A. tumefaciens, FtsZ-EGFP was detected at the new cell pole (15, 52). It is possible that in some bacteria, such as alphaproteobacteria, persistence of cell division proteins at the new cell pole after cell division might be a common feature, which is not related to the cell elongation mode.

### Conclusion.

Our survey and initial characterization of RgsP and RgsM interaction partners identified several novel components involved in unipolar cell growth and division in *Rhizobiales* and revealed links of these proteins to known cell wall growth and cell division factors. The newly identified Rgs proteins await a detailed analysis of their molecular functions and their interplay to unravel how the complex machineries of the elongasome and divisome accomplish cell growth and division in unipolarly growing alphaproteobacteria.

## MATERIALS AND METHODS

### Bacterial strains and growth conditions.

Bacterial strains and plasmids used in this study are shown in Table S1 in the supplemental material. S. meliloti was grown at 30°C in tryptone-yeast extract (TY) medium (53), LB medium (54), or modified morpholinepropanesulfonic acid (MOPS)-buffered minimal medium (55). When required, antibiotics were added to agar media at the following concentrations: streptomycin, 600 mg/liter; kanamycin, 200 mg/liter; gentamicin, 30 mg/liter; spectinomycin, 200 mg/liter. Unless otherwise indicated, IPTG was added to 500 μM. Alkaline phosphatase substrate 5-bromo-4-chloro-3-indolylphosphate (BCIP) was used at 50 μg/ml. E. coli was grown on LB at 37°C, and antibiotics were added at the following concentrations: kanamycin, 50 mg/liter; gentamicin, 8 mg/liter; spectinomycin, 100 mg/liter. For liquid cultures, antibiotic concentrations were reduced to one-half. IPTG was added to 100 μM. BCIP was used at 25 μg/ml.

10.1128/mBio.00306-20.4 TABLE S1 Strains and plasmids used in this study. Download Table S1, DOCX file, 0.1 MB. Copyright © 2020 Krol et al. 2020 Krol et al. This content is distributed under the terms of the Creative Commons Attribution 4.0 International license.

For growth assays involving protein depletion, bacteria were grown in 96-well plates in 100 μl of the medium with or without added IPTG in three replicates, in a Tecan M200PRO instrument, with alternating shaking (10 min) and nonshaking (20 min). Optical density was measured every 30 min. The precultures grown in TY medium with IPTG were used to inoculate the first cultures (culture I) with or without added IPTG to an optical density at 600 nm (OD_600_) of 0.005. After 16 h of growth, these cultures were diluted in the same media to an OD_600_ of 0.005 to start the second cultures (culture II), and growth curves were recorded for additional 40 h.

For growth assays involving gene overexpression, 3 independent transconjugant colonies were used to inoculate 100 μl of TY without IPTG in 96-well plates, followed by incubation with shaking at 1,200 rpm overnight. One microliter of these precultures was used to inoculate 100 μl of TY or LB medium with or without IPTG as indicated in the text, followed by incubation with shaking at 1,200 rpm for 16 h. Optical density was measured with a Tecan M200PRO instrument.

For fluorescence microscopy of liquid culture samples and for transmission electron microscopy, the S. meliloti strains were grown in 3 ml medium in glass tubes with shaking. Exponential-growth-phase samples were harvested at an OD_600_ between 0.4 and 0.8. For microscopy of protein depletion cultures, precultures in TY medium with added IPTG were used to inoculate TY cultures without added IPTG to an OD_600_ of 0.005 and grown for 20 to 24 h. For microscopy of gene overexpression strains, the precultures in TY medium without added IPTG were used to inoculate TY or LB medium with added IPTG (OD_600_ of 0.005 if the growth was not inhibited upon gene overexpression and OD_600_ of 0.01 to 0.05 if the growth was inhibited upon gene overexpression) and grown for 16 to 20 h. For determination of membrane topology using fusions to alkaline phosphatase, strains were grown in liquid cultures to stationary phase. Ten microliters of the S. meliloti cultures (10 μl of E. coli cultures diluted 1:10) were spotted onto TY (LB) agar plates with kanamycin, BCIP, and IPTG. Plates were photographed after 24 h of growth.

### Media.

Media used in this study are as follows: TY medium (5 g/liter tryptone, 3 g/liter yeast extract, 0.4 g CaCl_2_·2H_2_O), LB medium (10 g/liter tryptone, 5 g/liter yeast extract, 5 g/liter NaCl), and MOPS-buffered minimal medium (MM) (10 g/liter MOPS, 10 g/liter mannitol, 3.55 g/liter sodium glutamate, 0.246 g/liter MgSO_4_·7H_2_O, 0.25 mM CaCl_2_, 2 mM K_2_HPO_4_, 10 mg/liter FeCl_3_·6H_2_O, 1 mg/liter biotin, 3 mg/liter H_3_BO_3_, 2.23 mg/liter MnSO_4_·H_2_O, 0.287 mg/liter ZnSO_4_·7H_2_O, 0.125 mg/liter CuSO_4_·5H_2_O, 0.065 mg/liter CoCl_2_·6H_2_O, 0.12 mg/liter NaMoO_4_·2H_2_O, pH 7.2).

### Construction of strains and plasmids.

Cloning was performed using PCR, restriction digestion, ligation, and E. coli transformation. The strains and plasmids generated are listed in Table S1. Primers used in this study are shown in Table S2.

10.1128/mBio.00306-20.5 TABLE S2 Oligonucleotides used in this study. Download Table S2, DOCX file, 0.1 MB. Copyright © 2020 Krol et al. 2020 Krol et al. This content is distributed under the terms of the Creative Commons Attribution 4.0 International license.

To generate C-terminal fusions to mCherry or FLAG tag sequences encoded at the native genomic location, the C-terminal-portion-encoding sequence in the range of 500 to 800 bp was inserted in frame into the nonreplicative vectors pK18mob2-mCherry and pG18mob-CF, and the resulting plasmids were introduced by conjugation into S. meliloti strains. This resulted in positioning of the tagged gene copy under the control of the native promoter in the chromosome. To generate the strain with a markerless *mVenus-rgsS* fusion, the mVenus coding sequence was flanked by the upstream noncoding region and an N-terminal-portion-encoding sequence of the target gene. This construct was inserted into the sucrose selection plasmid pK18mobsacB. Double recombinants were selected on agar medium plates with sucrose (56).

To generate bacterial two-hybrid constructs, the corresponding coding sequences were inserted into the vectors pKT25, pUT18C, pKNT25Spe, and pUT18Spe in frame with a T25 or T18 fragment encoding sequences of Bordetella pertussis adenylate cyclase. Plasmid pKNT25Spe is a derivative of pKNT25, carrying a SpeI restriction site directly upstream of the protein-of-interest-T25 fusion translation start, which allows for preserving the native N terminus of the protein of interest. Plasmid pKNT25Spe was generated by PCR amplification of pKNT25 with primers listed in Table S2, restriction digestion with SpeI, and self-ligation.

To generate protein depletion strains, three different strategies were applied. To generate RgsD and RgsF depletion strains, the N-terminal-portion-encoding sequences, including 27 bp of the upstream noncoding sequence, were cloned into pK18mob2 in the orientation that resulted in placement of the *lac* promoter, carried by the vector, upstream of the partial *rgs* gene. Integration of these constructs into the genome by homologous recombination resulted in placement of the target gene under the control of the *lac* promoter. Subsequent introduction of pWBT, carrying *lacI*, resulted in IPTG-dependent expression of the target gene.

To generate RgsG, RgsH, TolQ, and Pal depletion strains, the corresponding gene was deleted from the genome using a pK18mobsac-based deletion construct and the sucrose selection procedure in the presence of an ectopic copy of the coding sequence of the gene of interest controlled by the *lac* promoter on plasmid pSRKGm, carrying *lacI*. This resulted in IPTG-dependent expression of the gene of interest.

To generate RgsA, RgsB, RgsC, RgsE, and RgsS depletion strains, the corresponding gene was deleted from the genome using a pK18mobsac-based deletion construct and the sucrose selection procedure in the presence of an ectopic gene copy including the native promoter on the single-copy curable plasmid pGCH14. This plasmid contains the replication operon *repABC* with a *lacI* box in the promoter region. Therefore, replication of this plasmid can be repressed by LacI. Subsequent introduction of pSRKKm, carrying *lacI*, resulted in IPTG-dependent replication of the complementation plasmid.

Gene overexpression plasmids were constructed by inserting the protein coding sequence into pWBT under the control of the IPTG-inducible T5 promoter.

The RgsD protein expression construct was generated using plasmid pWH844 containing sequences encoding an N-terminal His_6_ tag and RgsD lacking the predicted signal peptide.

### Fluorescence microscopy.

Microscopy was performed using the Nikon microscope Eclipse Ti-E equipped with a differential interference contrast (DIC) CFI Apochromat total internal-reflection fluorescence (TIRF) oil objective (100×; numerical aperture of 1.49) and a phase-contrast Plan Apo l oil objective (100×; numerical aperture, 1.45) with the AHF HC filter sets F36-513 DAPI (4′,6-diamidino-2-phenylindole) (excitation band pass [ex bp] 387/11 nm, beam splitter [bs] 409 nm, and emission [em] bp 447/60 nm), F36-504 mCherry (ex bp 562/40 nm, bs 593 nm, and em bp 624/40 nm), F36-525 EGFP (ex bp 472/30 nm, bs 495 nm, and em bp 520/35 nm), and F36-528 yellow fluorescent protein (YFP) (ex bp 500/24 nm, bs 520 nm, and em bp 542/27 nm). Images were acquired with an Andor iXon3 885 electron-multiplying charge-coupled-device (EMCCD) camera.

For microscopy of exponentially growing cultures, 2 μl of TY cultures at an OD_600_ of 0.4 to 0.8 were spotted onto 1% molecular biology-grade agarose (Eurogentec) pads, let dry for 2 to 3 min, covered with cover glass, and observed by microscopy. For time-lapse microscopy, bacteria from exponential-growth-phase TY cultures were diluted 1:20, and 2 μl was spread by gravity flow on the MM agarose pad and let dry for 14 min. The pads were covered air-tight with the cover slip and observed by microscopy in an incubation chamber at 30°C.

Cell morphology analysis was performed using the MicrobeJ plugin to ImageJ software. Graphical representation of the data was performed with the PRISM software (GraphPad).

### Transmission electron microscopy.

Sample preparation and microscopy were performed as described previously (12). Briefly, concentrated S. meliloti cell suspensions were high-pressure frozen and impregnated with substitute resin. The polymerized resin blocks containing the samples were cut to 50-nm-ultrathin sections using an ultramicrotome and applied onto 100 mesh copper grids coated with Pioloform. The sections were imaged using a JEM-2100 transmission electron microscope (JEOL, Tokyo, Japan) equipped with a 2k by 2kF214 fast-scan CCD camera (TVIPS, Gauting, Germany).

### Protein purification.

His_6_-RgsD was purified as described previously (57). Briefly, cells were lysed using M110-L microfluidizer (Microfluidics), and the protein was isolated using a 1-ml HisTrap column (GE Healthcare). Protein was concentrated with Amicon Ultracel-10K (Millipore) to a volume of 2 ml and applied to size exclusion chromatography (SEC) (HiLoad 26/600 Superdex 200 pg; GE Healthcare). After SEC, protein-containing fractions were pooled and concentrated with an Amicon Ultracel-10K (Millipore) according to the experimental requirements.

### Co-IP and protein identification by mass-spectrometry.

Coimmunoprecipitation (Co-IP) and protein identification by mass spectrometry were performed as previously described, including small modifications (58). Briefly, S. meliloti strains producing the C-terminal FLAG fusions and the negative-control strain Rm2011 carrying plasmid pWBT to confer resistance to gentamicin were grown in 200 ml of TY medium to an OD_600_ of 0.4 to 0.6, cross-linked with 0.36% formaldehyde for 15 min at room temperature (RT), and then quenched for 10 min with glycine (final concentration of 0.35 M). Cells were lysed using a French press instrument. Cleared lysates were obtained after centrifugation for 1 h at 20,000 × *g* and incubated with anti-FLAG M2 affinity gel (FLAG immunoprecipitation kit; Sigma). Bound proteins were eluted with 3×FLAG peptide solution.

Mass spectrometry analysis to identify eluted proteins was performed as described previously (12). For each pulldown sample, the total signal produced by peptides, matching S. meliloti proteins, was calculated as a sum of the area values of all detected proteins. For each detected protein, a fraction value was calculated as a percentage of the total signal. To calculate the relative protein enrichment, the fraction value was multiplied by the coverage value (percentage of the protein sequence covered by the identified peptides). The data were filtered to exclude proteins identified with less than two unique peptides. The data from experimental samples was further filtered to cut off proteins with a relative enrichment value of less than 0.5.

### Bacterial two-hybrid analysis.

Bacterial two-hybrid analysis was performed as previously described (59). The adenylate cyclase-deficient strain E. coli BTH101 was cotransformed with corresponding plasmid pairs. Single cotransformant colonies were inoculated in 100 μl LB supplemented with ampicillin and kanamycin and grown at 30°C for 6 h with shaking at 1,200 rpm. Ten microliters of each culture was spotted onto LB agar plates containing kanamycin, ampicillin, 40 mg/liter X-Gal, and 500 μM IPTG. Plates were imaged after 20 h of incubation at 30°C and 24 h of incubation at room temperature. Three independent cotransformant colonies were analyzed and produced similar results.

### Peptidoglycan purification and muropeptide analysis.

S. meliloti strains were grown in TY with IPTG overnight to obtain the precultures. These were used to inoculate 400 ml TY without IPTG to an OD_600_ of 0.001 (Rm2011 *rgsP-egfp*) or 0.005 (Rm2011 rgsP-*egfp rgsA*^dpl^) and grown for 20 h at 30°C. Isolation of PG sacculi was performed as described previously (12).

Purified sacculi (∼10 μg) were digested with the muramidase cellosyl, and the resulting muropeptides were analyzed by high-pressure liquid chromatography (HPLC) as previously described (12). The muropeptides were assigned according to established nomenclature (60).

### Peptidoglycan binding assay.

Purified PG (∼100 μg) from S. meliloti strain Rm2011 was centrifuged at 15,000 × *g* at 4°C for 14 min and resuspended in binding buffer (10 mM Tris-maleate, 10 mM MgCl_2_, 50 mM NaCl, pH 6.8). Ten micrograms of protein of interest was incubated with or without PG in a final volume of 100 μl and incubated at 4°C for 30 min. Samples were centrifuged as described above, and the supernatant was collected (supernatant fraction) while the pellet was resuspended in 200 μl of binding buffer. Another centrifugation step as described above was carried out (wash fraction). Bound proteins were released from PG by incubation with 100 μl of 2% SDS at 4°C for 1 h before being collected by a final centrifugation step as conducted at earlier steps. The proteins present in the different fractions were analyzed by SDS-PAGE.

### Homology search.

To assess the distribution of Rgs proteins in bacteria, 3,835 proteomes of the UniProt “Reference Proteomes 2019-05” set were searched (61). This subset does not include species with no or preliminary taxonomic classification (unclassified, *Candidatus* sp.) and species that represent subspecies of otherwise present species (genosp. and subsp.). The Rgs homologs from S. meliloti Rm2011, Agrobacterium tumefaciens (strain C58/ATCC 33970), Mesorhizobium loti MAFF303099, and Rhodopseudomonas palustris TIE-1 (exception: no RgsH and RgsS known in this species) were used as references.

For each Rgs protein, an iterative search was performed based on profile hidden Markov models of the reference sequences using jackhmmer (62). This initial search was limited to *Alphaproteobacteria* (552 species) and run with an E value threshold of 1e−40 and five iterations. Results were filtered automatically in two stages. The first stage was the size of the amino acid sequence, which should be within ±25% of the reference protein of S. meliloti 2011 (an exception is the large and repeat-containing RgsE protein for which a threshold of ±50% was used). The second stage was the annotation of transmembrane topology and signal peptides using the predictor tool Phobius (19). Assuming that the majority of putative homologs are correct, sequences with a transmembrane topology deviating from the most frequent one of the respective Rgs protein were removed. The remaining protein sequences were aligned progressively using MUSCLE (63) to create a largely improved-profile hidden Markov model. This model was then used in a single search run against proteomes of all bacterial classes using HMMsearch (64). MreB proteins were identified analogously except for an annotation and filtering step using Phobius. The dynamic cytoskeletal protein MreB from Escherichia coli (QGJ10319.1) was used as initial reference. MUSCLE-based alignments of all predicted homologs are shown in Data Set S5.

### Phylogeny of alphaproteobacterial families.

For each family of *Alphaproteobacteria*, one representative species was selected by choosing the species with the smallest taxonomy identifier (TaxID) in the UniProt reference proteomes. E. coli (TaxID 83333) was used in addition as an outgroup. The phylogenetic reconstruction was performed as described previously (65). The amino acid sequences of all 121 single-copy orthologs universal to this set identified by Proteinortho (66) with an E value of 1e−40 were aligned using MUSCLE and concatenated to a 57,845-amino-acid (aa) long “super protein.” Before this, C and N termini of the aligned sequences were trimmed until no gaps were left. ProtTest3 (67) identified the LG matrix with a gamma model of rate heterogeneity, an estimate of proportion of invariable sites and empirical base frequencies (+I+G+F) (68) as the best model according a Bayesian information criterion (BIC). Hence, a rapid bootstrap analysis with 1,000 replicates was performed to search for best-scoring maximum likelihood tree with respect to this model using RaxML with E. coli as outgroup (69).

### Data availability.

All coimmunoprecipitation data are presented in Data Set S1. Features from computational analysis of Rgs proteins are presented in Data Sets S2 and S3. Sequence comparisons underlying conservation analysis of Rgs proteins in *Alphaproteobacteria* are shown in Data Sets S4 and S5.
