# Supplementary figures and images for: Tol-Pal System and Rgs Proteins Interact to Promote Unipolar Growth and Cell Division in Sinorhizobium meliloti
Source: mBio. 2020 Jun 30;11(3):e00306-20. doi: 10.1128/mBio.00306-20 (PMC7327166; doi:10.1128/mBio.00306-20)

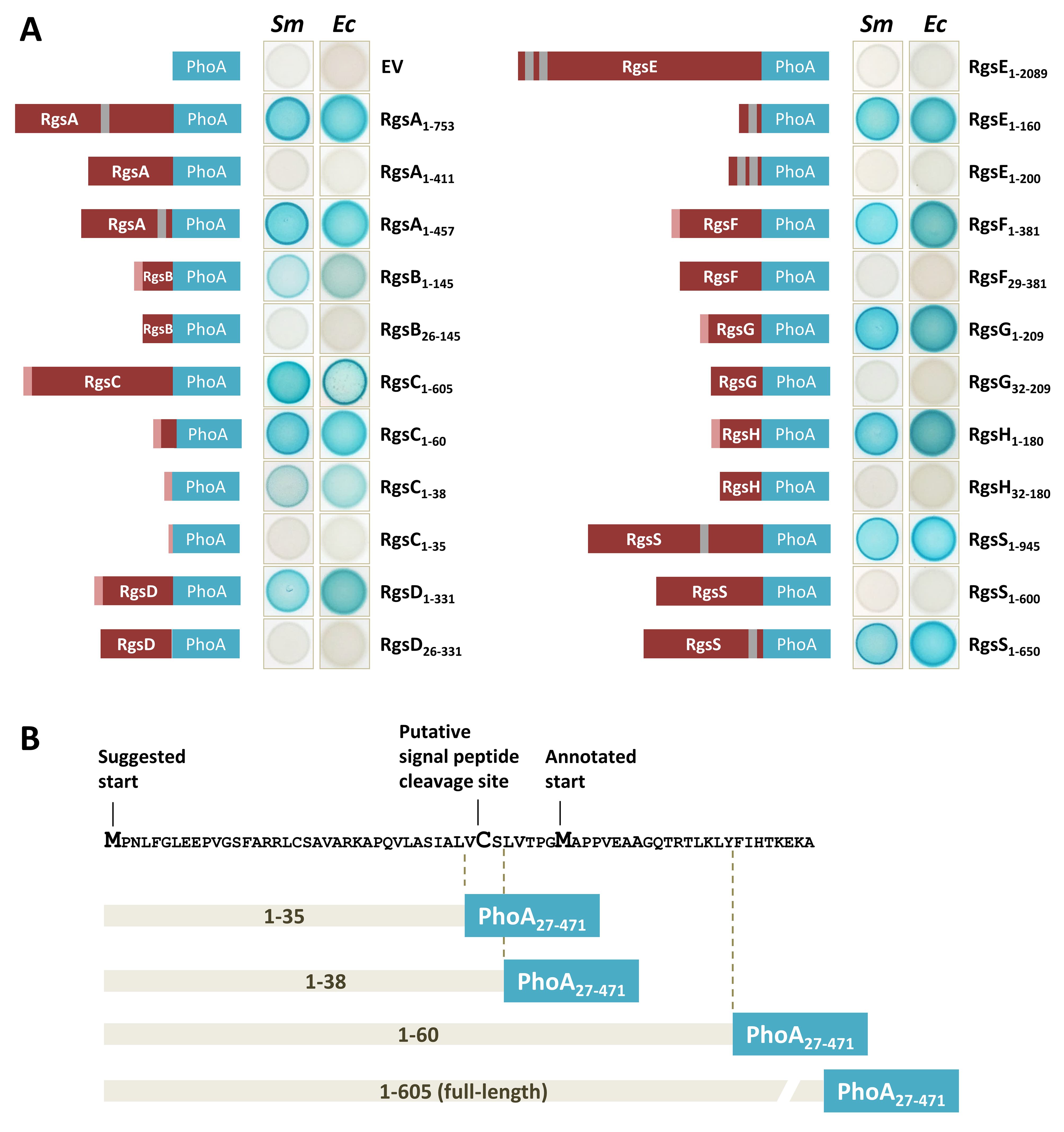

Supplement: FIG S1 [file mBio.00306-20-sf001.tif]

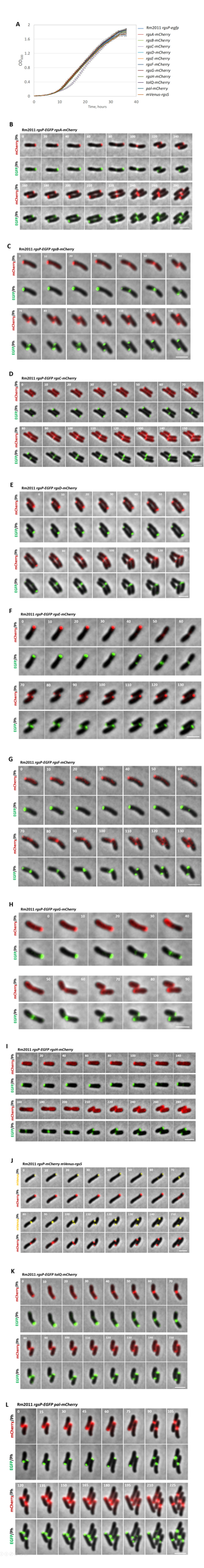

Supplement: FIG S2 [file mBio.00306-20-sf002.jpg]

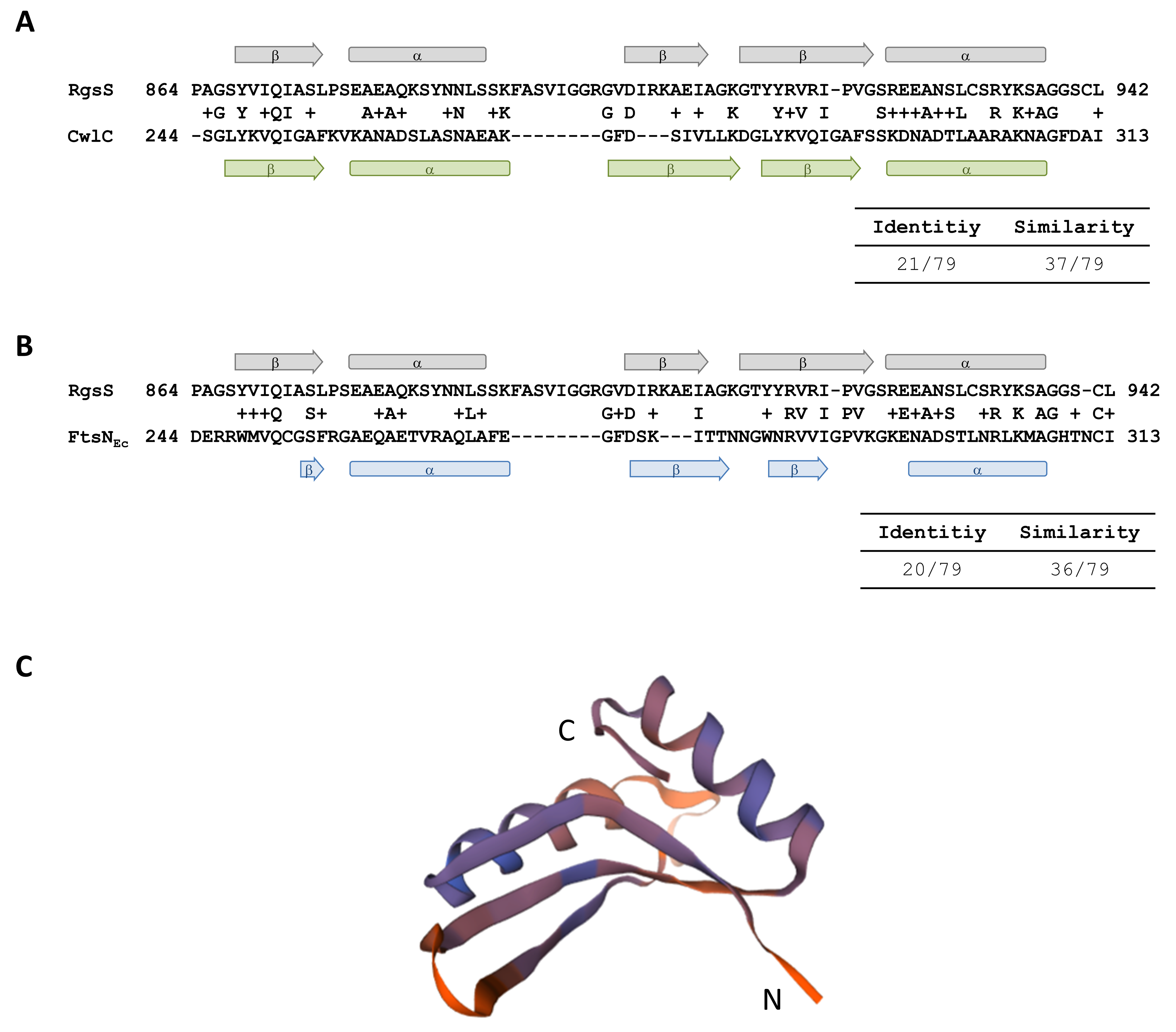

Supplement: FIG S3 [file mBio.00306-20-sf003.tif]
