# Supplementary material for: Tol-Pal System and Rgs Proteins Interact to Promote Unipolar Growth and Cell Division in Sinorhizobium meliloti
Source: mBio. 2020 Jun 30;11(3):e00306-20. doi: 10.1128/mBio.00306-20 (PMC7327166; doi:10.1128/mBio.00306-20)
Supplement: DATA SET S5 [file mBio.00306-20-sd005.pdf]

Dataset S5 - Alignment of Rgs proteins in α-proteobacteria - is available upon request.

https://hessenbox.uni-marburg.de/getlink/fiNZXWX9RVFRA7GPwtw1bsLB/Dataset_S5.pdf
