# Supplementary material for: Tol-Pal System and Rgs Proteins Interact to Promote Unipolar Growth and Cell Division in Sinorhizobium meliloti
Source: mBio. 2020 Jun 30;11(3):e00306-20. doi: 10.1128/mBio.00306-20 (PMC7327166; doi:10.1128/mBio.00306-20)
Supplement: TABLE S1 [file mBio.00306-20-st001.docx]

**Table S1. Strains and plasmids used in this study.**

| ***S. meliloti*** |  |  |
| --- | --- | --- |
| **Strain** | **Properties** | **Reference** |
| Rm2011 | Wild type, Str^r^ | 1 |
| Rm2011 *3*×*flag-mucR* | Rm2011 expressing *3*×*flag*-tagged *mucR*, markerless insertion | 2 |
| Rm2011 *rgsP-egfp* | Rm2011 expressing *egfp*-tagged *rgsP*, markerless insertion | 2 |
| Rm2011 *rgsP-mCherry* | Rm2011 expressing *mCherry*-tagged *rgsP*, markerless insertion | 2 |
| Rm2011 *rgsP-3*×*flag* | Rm2011 *rgsP*::pG18mob-*rgsP*-*3×flag*, Gm^r^ | 2 |
| Rm2011 *rgsM-3*×*flag* | Rm2011 *rgsM*::pG18mob-*rgsM*-*3×flag*, Gm^r^ | 2 |
| Rm2011 *rgsP-egfp rgsA-3*×*flag* | Rm2011 *rgsP-egfp rgsA*::pG18mob-*rgsA*-*3×flag*, Gm^r^ | This work |
| Rm2011 *rgsP-egfp rgsB-3*×*flag* | Rm2011 *rgsP-egfp rgsB*::pG18mob-*rgsB*-*3×flag*, Gm^r^ | This work |
| Rm2011 *rgsP-egfp rgsS-3*×*flag* | Rm2011 *rgsP-egfp rgsS*::pG18mob-*rgsS*-*3×flag*, Gm^r^ | This work |
| Rm2011 *rgsP-egfp tolQ-3*×*flag* | Rm2011 *egfp*-*rgsP*, carrying pSRKGm-tolQ-FLAG, markerless deletion of *tolQ,* Gm^r^ | This work |
| Rm2011 *rgsP-egfp pal-3*×*flag* | Rm2011 *rgsP-egfp pal*::pG18mob-*pal*-*3×flag*, Gm^r^ | This work |
| Rm2011 *rgsP-egfp rgsA-mCherry* | Rm2011 *rgsP-egfp rgsA*::pK18mob2-rgsA-mCherry, Km^r^ | This work |
| Rm2011 *rgsP-egfp rgsB-mCherry* | Rm2011 *rgsP-egfp rgsB*::pK18mob2-rgsB-mCherry, Km^r^ | This work |
| Rm2011 *rgsP-egfp rgsC-mCherry* | Rm2011 *rgsP-egfp rgsC*::pK18mob2-rgsC-mCherry, Km^r^ | This work |
| Rm2011 *rgsP-egfp rgsD-mCherry* | Rm2011 *rgsP-egfp rgsD*::pK18mob2-rgsD-mCherry, Km^r^ | This work |
| Rm2011 *rgsP-egfp rgsE-mCherry* | Rm2011 *rgsP-egfp rgsE*::pK18mob2-rgsE-mCherry, Km^r^ | This work |
| Rm2011 *rgsP-egfp rgsF-mCherry* | Rm2011 *rgsP-egfp rgsF*::pK18mob2-rgsF-mCherry, Km^r^ | This work |
| Rm2011 *rgsP-egfp rgsG-mCherry* | Rm2011 *rgsP-egfp rgsG*::pK18mob2-rgsG-mCherry, Km^r^ | This work |
| Rm2011 *rgsP-egfp rgsH-mCherry* | Rm2011 *rgsP-egfp rgsH*::pK18mob2-rgsH-mCherry, Km^r^ | This work |
| Rm2011 *mVenus-rgsS rgsP-mCherry* | Rm2011 expressing *mVenus*-tagged *rgsS* and *rgsP-mCherry*, markerless insertions | This work |
| Rm2011 *rgsP-egfp tolQ-mCherry* | Rm2011 *rgsP-egfp tolQ*::pK18mob2-tolQprCDS-mCherry, Km^r^ | This work |
| Rm2011 *rgsP-egfp pal-mCherry* | Rm2011 *rgsP-egfp pal*::pK18mob2-pal-mCherry, Km^r^ | This work |
| Rm2011 *rgsP-egfp rgsA^dpl^* | Rm2011 *egfp*-*rgsP*, carrying pGCH14-rgsA and pSRKKm, markerless deletion of *rgsA*, Km^r^, Gm^r^ | This work |
| Rm2011 *rgsP-egfp rgsB^dpl^* | Rm2011 *egfp*-*rgsP*, carrying pGCH14-rgsB and pSRKKm, markerless deletion of *rgsB*, Km^r^, Gm^r^ | This work |
| Rm2011 *rgsP-egfp rgsC^dpl^* | Rm2011 *egfp*-*rgsP*, carrying pGCH14-rgsC and pSRKKm, markerless deletion of *rgsC*, Km^r^, Gm^r^ | This work |
| Rm2011 *rgsP-egfp rgsD^dpl^* | Rm2011 *egfp*-*rgsP rgsD*::pK18m2-plac-rgsD carrying pWBT*,* Km^r^, Gm^r^ | This work |
| Rm2011 *rgsP-egfp rgsE^dpl^* | Rm2011 *egfp*-*rgsP*, carrying pGCH14-rgsE and pSRKKm, markerless deletion of *rgsE*, Km^r^, Gm^r^ | This work |
| Rm2011 *rgsP-egfp rgsE* | Rm2011 *egfp*-*rgsP*, carrying pSRKKm, markerless deletion of *rgsE*, Km^r^ | This work |
| Rm2011 *rgsP-egfp rgsF^dpl^* | Rm2011 *egfp*-*rgsP rgsF*::pK18m2-plac-rgsF carrying pWBT*,* Km^r^, Gm^r^ | This work |
| Rm2011 *rgsP-egfp rgsG^dpl^* | Rm2011 *egfp*-*rgsP*, carrying pSRKGm-rgsG, markerless deletion of *rgsG,* Gm^r^ | This work |
| Rm2011 *rgsP-egfp rgsH^dpl^* | Rm2011 *egfp*-*rgsP*, carrying pSRKGm-rgsH, markerless deletion of *rgsH,* Gm^r^ | This work |
| Rm2011 *rgsP-egfp rgsS^dpl^* | Rm2011 *egfp*-*rgsP*, carrying pGCH14-rgsS and pSRKKm, markerless deletion of *rgsS*, Km^r^, Gm^r^ | This work |
| Rm2011 *rgsP-egfp tolQ^dpl^* | Rm2011 *egfp*-*rgsP*, carrying pSRKGm-tolQ, markerless deletion of *tolQ,* Gm^r^ | This work |
| Rm2011 *rgsP-egfp pal^dpl^* | Rm2011 *egfp*-*rgsP*, carrying pSRKGm-pal, markerless deletion of *pal,* Gm^r^ | This work |
|  |  |  |
| ***E. coli*** |  |  |
| **Strain** | **Properties** | **Reference** |
| BL21(DE3) | F– *ompT gal dcm lon hsdSB*(*rB*–*mB*–) λ(DE3 [*lacI lacUV5*-*T7p07 ind1 sam7 nin5*]) [*malB*+]K-12(λS) | New England Biolabs |
| DH5α | F– *endA1 glnV44 thi-1 recA1 relA1 gyrA96 deoR nupG purB20* φ80d*lacZ*ΔM15 Δ(*lacZYA-argF*)U169, hsdR17(*rK*–*mK*+), λ– | 3 |
| S17-1 | *E. coli* 294 Thi RP4-2-Tc::Mu-Km::Tn7 integrated into the chromosome | 4 |
| BTH101 | *cya,* BACTH reporter strain | 5 |
|  |  |  |
| **Plasmids** |  |  |
| **Plasmid** | **Properties** | **Reference** |
| **Vectors** |  |  |
| pABC2Smob | Variant of mobilizable *repABC*-based mini-replicon, single-copy in *S. meliloti*, Spec^r^ | 6 |
| pABC-Psyn | pABC2Smob with added Kpn and NheI restriction sites, Spec^r^ | This work |
| pMlb-lacO | pK18mob2 carrying the repABC operon of pMLb carrying lacI box, Km^r^ | 6 |
| pGCH14 | pG18mob carrying the LacI-repressible repABC operon from pCH14, Gm^r^ | This work |
| pK18mob2 | suicide vector, *lacZ*, *mob*, Km^r^ | 7 |
| pK18mobsacB | suicide vector, *lacZ*, *mob*, *sacB,* Km^r^ | 7 |
| pSRKGm | pBBR1MCS-5-derived broad-host-range expression vector containing *lac* promoter and *lacI^q^*, *lacZα*^+^, Gm^r^ | 8 |
| pWBT | pSRKGm carrying T5 promoter, Gm^r^ | 9 |
| pK18mob2-mCherry | pK18mob2 carrying mCherry sequence, Km^r^ | This work |
| pG18mob-3×FLAG | pG18mob carrying 3×flag including a stop codon cloned into the XbaI and HindIII restriction sites, Gm^r^ | 2 |
| pWH844 | expression vector carrying 6×his and T5 promoter, Amp^r^ | 10 |
| pKT25 | Plasmid for constructing C-terminal fusions to T25, Km^r^ | 5 |
| pKNT25Spe | pKHT25 with a SpeI restriction site upstream of T25 start codon, Km^r^ | This work |
| pUT18Spe | pUT18 with a SpeI restriction site upstream of T18 start codon, Amp^r^ | 2 |
| pUT18C | Plasmid for constructing C-terminal fusions to T18, Amp^r^ | 5 |
| pUT18C-zip | pUT18C carrying the leucine zipper of GCN4, Amp^r^ | 5 |
| pKT25-zip | pKT25 carrying the leucine zipper of GCN4, Km^r^ | 5 |
|  |  |  |
| **BTH constructs** |  |  |
| pUT18Spe-rgsPΔGGDEFΔEAL | pUT18Spe carrying rgsP_1-524_ | 2 |
| pKNT25Spe-rgsPΔGGDEFΔEAL | pKNT25Spe carrying rgsP_1-524_ | 2 |
| pUT18C-rgsM | pUT18C carrying rgsM | 2 |
| pKT25-rgsM | pKT25 carrying rgsM | 2 |
| pKT25-rgsA | pKT25 carrying rgsA | This work |
| pUT18C-rgsA | pUT18C carrying rgsA | This work |
| pKNT25-rgsE | pKT25 carrying rgsE | This work |
| pUT18C-rgsE | pUT18Spe carrying rgsE | This work |
| pKT25-rgsS | pKT25 carrying rgsS | This work |
| pUT18C-rgsS | pUT18C carrying rgsS | This work |
| pKNT25Spe-tolQ | pKT25 carrying tolQ | This work |
| pUT18Spe-tolQ | pUT18Spe carrying tolQ | This work |
| pWH844-rgsD | pWH844 carrying rgsD_28-331_ | This work |
|  |  |  |
| **Overexpression constructs** |  |  |
| pWBT-rgsA | pWBT carrying *rgsA* coding sequence under lac-T5 promoter, Gm^r^ | This work |
| pWBT-rgsB | pWBT carrying *rgsB* coding sequence under lac-T5 promoter, Gm^r^ | This work |
| pWBT-rgsC | pWBT carrying *rgsC* coding sequence under lac-T5 promoter, Gm^r^ | This work |
| pWBT-rgsD | pWBT carrying *rgsD* coding sequence under lac-T5 promoter, Gm^r^ | This work |
| pWBT-rgsE | pWBT carrying *rgsE* coding sequence under lac-T5 promoter, Gm^r^ | This work |
| pWBT-rgsF | pWBT carrying *rgsF* coding sequence under lac-T5 promoter, Gm^r^ | This work |
| pWBT-rgsG | pWBT carrying *rgsG* coding sequence under lac-T5 promoter, Gm^r^ | This work |
| pWBT-rgsH | pWBT carrying *rgsH* coding sequence under lac-T5 promoter, Gm^r^ | This work |
| pWBT-rgsS | pWBT carrying *rgsS* coding sequence under lac-T5 promoter, Gm^r^ | This work |
| pWBT-tolQ | pWBT carrying *tolQ* coding sequence under lac-T5 promoter, Gm^r^ | This work |
|  |  |  |
| **Ectopic expression constructs** |  |  |
| pGCH14-rgsA | pGCH14 carrying *rgsA* promoter and coding sequence, Spec^r^ | This work |
| pGCH14-rgsB | pGCH14 carrying *rgsB* promoter and coding sequence, Spec^r^ | This work |
| pGCH14-rgsC | pGCH14 carrying *rgsC* promoter and coding sequence, Spec^r^ | This work |
| pGCH14-rgsE | pGCH14 carrying *rgsE* promoter and coding sequence, Spec^r^ | This work |
| pGCH14-rgsS | pGCH14 carrying *rgsS* promoter and coding sequence, Spec^r^ | This work |
| pSRKGm-rgsG | pSRKGm carrying *rgsG* coding sequence under lac promoter, Gm^r^ | This work |
| pSRKGm-rgsH | pSRKGm carrying *rgsH* coding sequence under lac promoter, Gm^r^ | This work |
| pSRKGm-tolQ | pSRKGm carrying *tolQ* coding sequence under lac promoter, Gm^r^ | This work |
| pSRKGm-tolQ-CF | pSRKGm carrying *tolQ* coding sequence fused to 3xFLAG tag sequence under lac promoter, Gm^r^ | This work |
| pSRKGm-pal | pSRKGm carrying pal coding sequence under lac promoter, Gm^r^ | This work |
| pSRKKm-phoA | pSRKKm carrying E. coli PhoA_27-471_ coding sequence | This work |
| pSRKKm-rgsA-phoA | pSRKKm-phoA carrying RgsA coding sequence | This work |
| pSRKKm-rgsA_1-411_-phoA | pSRKKm-phoA carrying RgsA_1-411_ coding sequence | This work |
| pSRKKm-rgsA_1-457_-phoA | pSRKKm-phoA carrying RgsA_1-457_ coding sequence | This work |
| pSRKKm-rgsB-phoA | pSRKKm-phoA carrying RgsB coding sequence | This work |
| pSRKKm-rgsB_26-145_-phoA | pSRKKm-phoA carrying RgsB_26-145_ coding sequence | This work |
| pWBT-rgsC-phoA | pWBT carrying RgsC and PhoA_27-471_ coding sequences | This work |
| pSRKKm-rgsC_1-35_-phoA | pSRKKm-phoA carrying RgsC_1-35_ coding sequence | This work |
| pSRKKm-rgsC_1-38_-phoA | pSRKKm-phoA carrying RgsC_1-38_ coding sequence | This work |
| pSRKKm-rgsC_1-60_-phoA | pSRKKm-phoA carrying RgsC_1-60_ coding sequence | This work |
| pSRKKm-rgsD-phoA | pSRKKm-phoA carrying RgsD coding sequence | This work |
| pSRKKm-rgsD_26-331_-phoA | pSRKKm-phoA carrying RgsB_26-331_ coding sequence | This work |
| pSRKKm-rgsE-phoA | pSRKKm-phoA carrying RgsE coding sequence | This work |
| pSRKKm-rgsE_1-160_-phoA | pSRKKm-phoA carrying RgsE_1-160_ coding sequence | This work |
| pSRKKm-rgsE_1-200_-phoA | pSRKKm-phoA carrying RgsE_1-200_ coding sequence | This work |
| pSRKKm-rgsF-phoA | pSRKKm-phoA carrying RgsF coding sequence | This work |
| pSRKKm-rgsF_29-381_-phoA | pSRKKm-phoA carrying RgsF_29-381_ coding sequence | This work |
| pSRKKm-rgsG-phoA | pSRKKm-phoA carrying RgsG coding sequence | This work |
| pSRKKm-rgsG_32-209_-phoA | pSRKKm-phoA carrying RgsG_32-209_ coding sequence | This work |
| pSRKKm-rgsH-phoA | pSRKKm-phoA carrying RgsH coding sequence | This work |
| pSRKKm-rgsH_32-180_-phoA | pSRKKm-phoA carrying RgsH_32-180_ coding sequence | This work |
| pSRKKm-rgsS-phoA | pSRKKm-phoA carrying RgsS coding sequence | This work |
| pSRKKm-rgsS_1-600_-phoA | pSRKKm-phoA carrying RgsS_1-600_ coding sequence | This work |
| pSRKKm-rgsS_1-650_-phoA | pSRKKm-phoA carrying RgsS_1-650_ coding sequence | This work |
|  |  |  |
| **Integrative plasmids** |  |  |
| pK18mob2-plac-rgsD | pK18mob2carrying 27 bp of the *rgsD* upstream non-coding sequence and a 5’ portion of the coding sequence, Km^r^ | This work |
| pK18mob2-plac-rgsF | pK18mob2carrying 27 bp of the *rgsF* upstream non-coding sequence and a 5’ portion of the coding sequence, Km^r^ | This work |
| pK18mobsacB-rgsAdel | pK18mobsacB carrying *rgsA* flanking regions, Km^r^ | This work |
| pK18mobsacB-rgsBdel | pK18mobsacB carrying *rgsB* flanking regions, Km^r^ | This work |
| pK18mobsacB-rgsCdel | pK18mobsacB carrying *rgsC* flanking regions, Km^r^ | This work |
| pK18mobsacB-rgsEdel | pK18mobsacB carrying *rgsE* flanking regions, Km^r^ | This work |
| pK18mobsacB-rgsGdel | pK18mobsacB carrying *rgsG* flanking regions, Km^r^ | This work |
| pK18mobsacB-rgsHdel | pK18mobsacB carrying *rgsH* flanking regions, Km^r^ | This work |
| pK18mobsacB-rgsSdel | pK18mobsacB carrying *rgsS* flanking regions, Km^r^ | This work |
| pK18mobsacB-tolQdel | pK18mobsacB carrying *tolQ* flanking regions, Km^r^ | This work |
| pK18mobsacB-paldel | pK18mobsacB carrying *pal* flanking regions, Km^r^ | This work |
| pK18mob2-rgsA-mCherry | pK18mob2-mCherry carrying 3' portion of *rgsA*, Km^r^ | This work |
| pK18mob2-rgsB-mCherry | pK18mob2-mCherry carrying 3' portion of *rgsB*, Km^r^ | This work |
| pK18mob2-rgsC-mCherry | pK18mob2-mCherry carrying 3' portion of *rgsC*, Km^r^ | This work |
| pK18mob2-rgsD-mCherry | pK18mob2-mCherry carrying 3' portion of *rgsD*, Km^r^ | This work |
| pK18mob2-rgsE-mCherry | pK18mob2-mCherry carrying 3' portion of *rgsE*, Km^r^ | This work |
| pK18mob2-rgsF-mCherry | pK18mob2-mCherry carrying 3' portion of *rgsF*, Km^r^ | This work |
| pK18mob2-rgsG-mCherry | pK18mob2-mCherry carrying 3' portion of *rgsG*, Km^r^ | This work |
| pK18mob2-rgsH-mCherry | pK18mob2-mCherry carrying 3' portion of *rgsH*, Km^r^ | This work |
| pK18mob2-tolQ-prCDS-mCherry | pK18mob2-mCherry carrying 3' portion *tolQ* coding sequence and promoter region, Km^r^ | This work |
| pK18mob2-pal-mCherry | pK18mob2-mCherry carrying 3' portion of *pal*, Km^r^ | This work |
| pK18mobsacB-*mVenus-rgsS* | pK18mobsacB carrying 5' portion of *rgsS* excluding the start codon, *mVenus* and *rgsS* upstream region, Km^r^ | This work |
| pG18mob-rgsA-3*×flag* | pG18mob-*3×flag* carrying 3' portion of *rgsA*, Gm^r^ | This work |
| pG18mob-rgsB-3*×flag* | pG18mob-*3×flag* carrying 3' portion of *rgsB*, Gm^r^ | This work |
| pG18mob-pal-3*×flag* | pG18mob-*3×flag* carrying 3' portion of *pal*, Gm^r^ | This work |
| pG18mob-rgsS-3*×flag* | pG18mob-*3×flag* carrying 3' portion of *rgsS*, Gm^r^ | This work |

**References**

1. Casse F, Boucher C, Julliot J, Michel M, Dénarié J. 1979. Identification and characterization of large plasmids in *Rhizobium meliloti* using agarose gel electrophoresis. Microbiology 113:229-242.
2. Schäper S, Yau HCL, Krol E, Skotnicka D, Heimerl T, Gray J, Kaever V, Søgaard-Andersen L, Vollmer W, Becker A. 2018. Seven-transmembrane receptor protein RgsP and cell wall-binding protein RgsM promote unipolar growth in Rhizobiales. PLoS Genet 14:e1007594.
3. Grant SG, Jessee J, Bloom FR, Hanahan D. 1990. Differential plasmid rescue from transgenic mouse DNAs into *Escherichia coli* methylation-restriction mutants. Proc Natl Acad Sci U S A 87:4645-4649.
4. Simon R, Priefer U, Pühler A. 1983. A broad host range mobilization system for *in vivo* genetic engineering: transposon mutagenesis in Gram-negative bacteria. Nature Biotechnology 1:784-791.
5. Karimova G, Pidoux J, Ullmann A, Ladant D. 1998. A bacterial two-hybrid system based on a reconstituted signal transduction pathway. Proc Natl Acad Sci U S A 95:5752-5756.
6. Döhlemann J, Wagner M, Happel C, Carrillo M, Sobetzko P, Erb TJ, Thanbichler M, Becker A. 2017. A family of single copy repABC-type shuttle vectors stably maintained in the alpha-proteobacterium *Sinorhizobium meliloti*. ACS Synth Biol. 6:968-984.
7. Schäfer A, Tauch A, Jäger W, Kalinowski J, Thierbach G, Pühler A. 1994. Small mobilizable multi-purpose cloning vectors derived from the *Escherichia coli* plasmids pK18 and pK19: selection of defined deletions in the chromosome of *Corynebacterium glutamicum*. Gene 145:69-73.
8. Khan SR, Gaines J, Roop RM, Farrand SK. 2008. Broad-host-range expression vectors with tightly regulated promoters and their use to examine the influence of TraR and TraM expression on Ti plasmid quorum sensing. Appl Environ Microbiol 74:5053-5062.
9. Schlüter JP, Czuppon P, Schauer O, Pfaffelhuber P, McIntosh M, Becker A. 2015. Classification of phenotypic subpopulations in isogenic bacterial cultures by triple promoter probing at single cell level. J Biotechnol. 198:3-14.
10. Schirmer F, Ehrt S, Hillen W. 1997. Expression, inducer spectrum, domain structure, and function of MopR, the regulator of phenol degradation in *Acinetobacter calcoaceticus* NCIB8250. J Bacteriol 179:1329-1336.
