# Supplementary material for: Tol-Pal System and Rgs Proteins Interact to Promote Unipolar Growth and Cell Division in Sinorhizobium meliloti
Source: mBio. 2020 Jun 30;11(3):e00306-20. doi: 10.1128/mBio.00306-20 (PMC7327166; doi:10.1128/mBio.00306-20)
Supplement: TABLE S2 [file mBio.00306-20-st002.docx]

**Table S2.** Oligonucleotides used in this study

| **Oligonucleotide** | **Sequence** | **Cloning destination** |
| --- | --- | --- |
| Smc00644+1762-Hind-f | gaacaagcttACCGTTGCCTGGTCGATCAAG | C-terminal mCherry fusion plasmids |
| Smc00644-endonostop-Xba-r | tcgtctagaCTGCCCGCTGGAAGCTGTGCT |  |
| Smc04006+48-Hind-f | cgtaaagcTTTCTCTCTGCCGGCGATGC |  |
| Smc04006-endnostop-Xba-r | atatctagaATTCTGGCTTTCCTGCGTGCC |  |
| Smc04010-mCh+1299-Hind-f | ccttaagctTCACGCCGAGGTCACTATTCC |  |
| Smc04010-endnostop-Xba-r | cactctagaGTTTGTGCTGAACTTCTTCAC |  |
| Smc01011+463+-Hind-f | cctcaagcttGTCATGCTGAACGCCGTCTCC |  |
| Smc01011endonostop-Xba-r | gtctctagaGTCCTGCGCTTCCGCGGCCTG |  |
| Smc00190-mCh+5800-Hind-f | gaagaagcttCAACGGAGGAACGTGCCGAAG |  |
| Smc00190_endnostop-Xba-r | tgctctagaGCTGAAGCGCCCTGCCGCGT |  |
| Smc03995+629-Hind-f | atataagcttAGTTCAACTACGGGCAAACGC |  |
| Smc03995_endnostop-Xba-r | cgttctagaGGTCGAGCGATACTTGTTTGC |  |
| Smc00950+151-Hind-f | ctgcaagcttGACAACGATGTCTGCATCGTC |  |
| Smc00950endnostop-Xba-r | gtatctagaCTGCTGCTTGGCAGCCTTTTG |  |
| Smc00153+151-Sal-f | caaggtcgacTTCGACAACATTCTGCCGAAC |  |
| Smc00153_endnostop_Xba-r | gtttctagaTTGGGCGGGCGCCGCAGCGCCTGCATTTG |  |
| tolQ-486-SphI-f | ccagcatgcGTCGCAGCGTGCTTCTGAACC |  |
| tolQ-nostop-Xba-r | gaattctagaTTGCGCGGCTTGGCGAGAA |  |
| pal+112-Hind-f | ccgtaagcttAACCTGCCGAACGATGCTGCC |  |
| pal-endnostop-Xba-r | cactctagaGCTGCCGGCGCCACCGAGCAC |  |
| 02072+3-Kpn-r | atatggtaccCATACTCTCCACTCACAACTG | mVenus-RgsS fusion plasmid |
| Smc02072+750-Hind-r | atataagcttTGACGGTTGCGACGGCAGTTC |  |
| **Table S2. Continued** | |  |
| EGFP/mCh+1-f-Kpn | atatggtaccATGGTGAGCAAGGGCGAGGAG | mVenus-RgsS fusion plasmid |
| Smc02072del1-Eco-f | atatgaattcGAAGCTGACGGTGCTGCTCTG |  |
| Smc02072+4-Xba-f | agcatctagaGCAGACAAACAATTCGCACGA |  |
| EGFP/mCh-Xba-r-nostop | gtactctagaCTTGTACAGCTCGTCCATG |  |
| Smc01011-27-Xba-f | tcatctagaCACCCAGTCAAGAAGTCAACT | Depletion plasmids |
| Smc01011+482-Hind-r | gcagaagcttGAGACGGCGTTCAGCATGACG |  |
| Smc03995-27-Xba-f | gactctagaCCACGGTTTGAAGGTTAAGAC |  |
| Smc03995+470-Hind-r | caacaagcttGCCTTGGCGTCGCGTGCAAC |  |
| Smc00644del1-Sal-f | tatagtcgacTCATGGACGAGCTGTCGTCGG | Deletion plasmids |
| Smc00644del1-Xba—r | aactctagaCAATTTCGAGACCCGCGGCAA |  |
| Smc00644del2-Xba—f | tattctagAGCACTTCCCGGAAAAGTGGG |  |
| Smc00644del2—Eco-r | atatgaattcTGCAGGCGGTCAAGAAGTTCG |  |
| Smc04006del1-Eco-f | tgtagaattCGTTGTTGCCGACGACGATGT |  |
| Smc04006del1-Xba-r | tcctctaGATATCTAGACACGGCTTCCT |  |
| Smc04006del2-Xba-f | acgtctagAGCTTTCCGACACGCCCGGCA |  |
| Smc04006del2-Hind-r | gtagaagcttAGCGCATCCGAAAGCTCGATG |  |
| Smc04010del1-Eco-f | cttggaattcGCAAGGAGGATATCCCCGTTC |  |
| Smc04010del1-Xba-r | acatctagaTCAATCCCCGTCCGACGGACA |  |
| Smc04010del2-Xba-f | caatctagACCTTCGCTTGCCGCCTAAAC |  |
| Smc04010del2-Hind-r | cgacaagcttACACCGATGAGCGAAATCAGC |  |
| 00190del1-Eco-f | atatgaattCAAGGTCGATGGGATTCTTGC |  |
| 00190del1-Xba-r | tattctagaCTACTATTGCCCGATGATGGC |  |
| 00190del2-Xba-f | atatctagACACGCAATCGGTCTCTCGGG |  |
| **Table S2. Continued** | |  |
| 00190del2-Hind-r | atataagcTTTCATGGCTACGGCATGCAC | Deletion plasmids |
| Smc00950del1-Hind-f | ctgtaagcttAAAGGCGCACGCCCTTTGTC |  |
| Smc00950del1-Xba-f | caatctagaCCTGAAATCCGGTCACGGAAC |  |
| Smc00950del2-Xba-f | gaatctagaCATTAGCTTCTCTGAGTAAAG |  |
| Smc00950del2-Eco-r | tattgaaTTCGACATGTACTATTCCGTC |  |
| Smc00153del1-Hind-f | gacaaagctTGCGGCTTGGAATAGGCACAG |  |
| Smc00153del1-Xba-r | gtgtctagATGCTCCTGTGTTGGCACGTG |  |
| Smc00153del2-Xba-f | caatctagaCCAATAAGCCCTCGGGCGAAC |  |
| Smc00153del2-Eco-r | ggttgaattcGAAGATCGAAGGCTTCGTTGG |  |
| Smc02072del1-Eco-f | atatgaattcGAAGCTGACGGTGCTGCTCTG |  |
| Smc02072del1-Xba-r | tattctagaCACAACTGCCTGCGTTGAATTG |  |
| SMc02072del2-X-f2 | ccttctagaCGTATTACCGGGTCAGGATCC |  |
| Smc02072del2-H-r3 | atataagcttGGCATGTGCTTCATCACCGG |  |
| tolQdel1-Eco-f | gttcgaattcTGCAGCTTGAAGGAGAGCTTG |  |
| tolQdel1-Xba-r | gactctagaCCGAATCCAAACACCCGGCAG |  |
| tolQdel2-Xba-f | ccatctagaCGACCTCAAAGCACGGAGACC |  |
| tolQdel2-Hind-r | cttgaagcttGTCTTCAGGTCGACGTCGTTC |  |
| pal_del1-Eco-f | cgctgaattcACACCTCTCCCTCCTATTCGC |  |
| pal_del1-Xba-r | ctgtctagaCGGGTCTCCTTGAGAAGTGTC |  |
| pal_del2-Xba-f | gattctagaGGCAGCTGATTATTCGGCATAG |  |
| pal_del2-Hind-r | tataaagcttCTCGTCGAAAATGATCTGCCC |  |
| Smc00644-432-Xba-f | atatctagaTGGTTCGATGCTGCGCTGG | Complementation plasmids |
| Smc00644end-Nco-r | tgtcccatggCTACTGCCCGCTGGAAGCTGT |  |
| **Table S2. Continued** | |  |
| PSMc04006_Eco_fwd | ATATGAATTCTGGTCGGCAACCAGCACGA | Complementation plasmids |
| SMc04006_Mlu_rev | ATATACGCGTTCAATTCTGGCTTTCCTGCGT |  |
| Smc04010-400-Spe | atatactagtGGAAGGCCGTCTGGAGAACGC |  |
| Smc04010-stop-Nco-r | cactccatggTCAGTTTGTGCTGAACTTCTTCAC |  |
| SMc00190-500-Xba-f | atatctagaGCACACACTTTCCTCATCCCG |  |
| SMc00190stop-Not-r | atatgcggccgcTCAGCTGAAGCGCCCTGCCGCGT |  |
| Smc00950+1-Nde-f | cactcatATGATCTTCAAGTCGAACTTCAC |  |
| Smc00950-stop-Kpn-r | ttccggtaccTTACTGCTGCTTGGCAGCCTT |  |
| Smc00153+1-Nde-f | atatcatATGAACAAATTCGCAGGTCTTGC |  |
| Smc00153stop-Kpn-r | tataggtaccGTTCGCCCGAGGGCTTATTGG |  |
| Smc02072-400-Xba-f | atatctagaTCTTGATCTGTCCTCAGTCG |  |
| Smc02072-stop-Nco-r | ccttccatggttATTTCGTTACCAGGCAGCTTCC |  |
| tolQ+1-Nde-f | tattcatATGGAACAGGTTGGATTGGCC |  |
| tolQ-stop-Kpn-r | gagcggtaccTTATTGCGCGGCTTGGCGAG |  |
| pal+1-Nde-f | gctacatATGAGCCGAATTGACACCCCG |  |
| pal_stop-Kpn-r | ggtaccTCAGCTGCCGGCGCCACCGAG |  |
| Smc00644+1-Xba-f | ttgtctagaATGGCGGATTTTGTTGCAGTT | Overexpression plasmids |
| Smc00644end-Kpn-r | tgtcggtacCTACTGCCCGCTGGAAGCTGT |  |
| Smc04006+1-Xba-f | ctctctagaATGACGTTTGGATCCATCCTC |  |
| Smc04006_stop-Hind-r | gtgcaagctTCAATTCTGGCTTTCCTGCGTG |  |
| Smc01011+1-Xba-f | ttatctagaATGCGTCTCCGTCTTCGTATT |  |
| Smc01011stop-Kpn-r | agtcggtaccTCAGTCCTGCGCTTCCGCG |  |
| Smc00190+1-Xba-f | caatctagaATGGCGACGAAGAAGACCAAC |  |
| **Table S2. Continued** | |  |
| Smc00190_stop-Kpn-r | tgcggtaccTCAGCTGAAGCGCCCTGCCGCGT | Overexpression plasmids |
| Smc03995+1-Xba-f | gtctctagaATGGTAATCCGCTCTCTCCTG |  |
| Smc03995_stop-Kpn-r | ttgtggtaccTCAGGTCGAGCGATACTTGTT |  |
| Smc00950+1-Xba-f | cactctagaATGATCTTCAAGTCGAACTTCAC |  |
| Smc00950-stop-Kpn-r | ttccggtaccTTACTGCTGCTTGGCAGCCTT |  |
| SMc00153+1-Xba-f | atatctagaATGAACAAATTCGCAGGTCTTGC |  |
| Smc00153stop-Kpn-r | tataggtaccGTTCGCCCGAGGGCTTATTGG |  |
| Smc02072+1-Xba-f | cactctagaATGGCAGACAAACAATTCGCAC |  |
| Smc02072-stop-Kpn-r | cgacggtaccGTTTATTTCGTTACCAGGCAGC |  |
| tolQ-stop-Kpn-r | gagcggtaccTTATTGCGCGGCTTGGCGAG |  |
| tolQ+1-Xba-f | tattctagaATGGAACAGGTTGGATTGGCC |  |
| Smc00644+1-Xba-g-f | ttgtctagagATGGCGGATTTTGTTGCAGTT | Bacterial two-hybrid plasmids |
| Smc00644end-Kpn-r | tgtcggtacCTACTGCCCGCTGGAAGCTGT |  |
| Smc02072+3-Xba-f | agcatctagaGGCAGACAAACAATTCGCACG |  |
| Smc02072-stop-Kpn-r | cgacggtaccGTTTATTTCGTTACCAGGCAGC |  |
| Smc00190+3-Xba-f | caatctagaGGCGACGAAGAAGACCAACGA |  |
| Smc00190_stop-Kpn-r | tgcggtaccTCAGCTGAAGCGCCCTGCCGCGT |  |
| tolQ+1-Xba-f | tattctagaATGGAACAGGTTGGATTGGCC |  |
| tolQ-nostop-Kpn-r | gaatggtaccTGCGCGGCTTGGCGAGAAG |  |
| pCH14-BamNcoNotPacMluSpe-r | atatggatccatggcggccgcttaattaacgcgtactagtGGCGTTCGTCACTCGTTTGGC | pGCH14 |
| pCH14-Eco-f | TTACGAATTCGAGCTCGGTAC |  |
| Smc01011+82-Bam-f | agaaggatccGATCCGACTGACGCCTTCAAGA | RgsD protein expression plasmid |
| Smc01011stop-Pst-r | agtcctgcagTCAGTCCTGCGCTTCCGCG |  |
| **Table S2. Continued** | |  |
| Smc00644+1762-Eco-f | gaacgaattcACCGTTGCCTGGTCGATCAAG | C-terminal FLAG fusion plasmids |
| Smc00644-endonostop-Xba-r | tcgtctagaCTGCCCGCTGGAAGCTGTGCT |  |
| Smc04006-endnostop-Xba-r | atatctagaATTCTGGCTTTCCTGCGTGCC |  |
| Smc04006+48-Eco-f | cgtagaaTTCTCTCTGCCGGCGATGC |  |
| Smc02072_endnostopXba-r | ccttctagaTTTCGTTACCAGGCAGCTTCC |  |
| Smc02072+1867-Eco—f | atatgaattcGGCAGCAACGCGGTGCTTTCC |  |
| tolQ+1-Nde-f | tattcatATGGAACAGGTTGGATTGGCC |  |
| tolQ-nostop-Xba-r | gaattctagaTTGCGCGGCTTGGCGAGAA |  |
